# APC and ZBTB2 May Mediate M2 Macrophage Infiltration to Promote the Development of Renal Fibrosis: A Bioinformatics Analysis

**DOI:** 10.1155/2024/5674711

**Published:** 2024-09-18

**Authors:** Jianling Song, Ben Ke, Xiangdong Fang

**Affiliations:** Department of Nephrology The Second Affiliated Hospital Jiangxi Medical College Nanchang University, Nanchang, Jiangxi 330006, China

**Keywords:** biomarker, chronic kidney disease, immune infiltration, renal fibrosis, uremia

## Abstract

**Background and Purpose:** The continuous accumulation of M2 macrophages may potentially contribute to the development of kidney fibrosis in chronic kidney disease (CKD). The purpose of this study was to analyze the infiltration of M2 macrophages in uremic patients and to seek new strategies to slow down the progression of renal fibrosis.

**Methods:** We conducted a comprehensive search for expression data pertaining to uremic samples within the Gene Expression Omnibus (GEO) database, encompassing the time frame from 2010 to 2022. Control and uremic differentially expressed genes (DEGs) were identified. Immune cell infiltration was investigated by CIBERSORT and modules associated with M2 macrophage infiltration were identified by weighted gene coexpression network analysis (WGCNA). Consistent genes were identified using the least absolute shrinkage and selection operator (LASSO) and selection and visualization of the most relevant features (SVM-RFE) methods to search for overlapping genes. Receiver operating characteristic (ROC) curves were examined for the diagnostic value of candidate genes. Quantitative real-time PCR (qPCR) examined the expression levels of candidate genes obtained from uremic patients in M2 macrophage.

**Results:** A total of 1298 DEGs were identified within the GSE37171 dataset. Significant enrichment of DEGs was observed in 20 biological processes (BP), 19 cellular components (CC), 6 molecular functions (MF), and 70 Kyoto Encyclopedia of Genes and Genomes (KEGG) pathways. CIBERSORT analysis observed a significant increase in B-cell memory, dendritic cell activation, M0, M1, M2, and plasma cell numbers in uremic samples. We identified the 10 most interrelated genes. In particular, adenomatous polyposis coli (APC) and zinc finger and BTB structural domain 2 (ZBTB2) were adversely associated with the infiltration of M2 macrophages. Importantly, the expression levels of APC and ZBTB2 were far lower in M2 macrophages from uremic patients than those in healthy individuals.

**Conclusion:** The development of renal fibrosis may be the result of M2 macrophage infiltration promoted by APC and ZBTB2.

## 1. Introduction

The prevalence of people with chronic kidney disease (CKD) has been rising, affecting an estimated 843.6 million people worldwide in 2017, partly due to a gradual increase in risk factors, including obesity and diabetes [1]. CKD will be the fifth most common cause of death worldwide by 2040 [2]. Due to the high morbidity and mortality of CKD, finding strategies to slow down the course of CKD is crucial for patients, society, and the nation.

Activation of mesenchymal cells and fibroblasts, epithelial-mesenchymal transition of renal tubules, and monocyte/macrophage infiltration and apoptosis are a series of biological processes (BP) leading to the development of fibrosis [3–5]. Specifically, M2 macrophage infiltration is an important factor contributing to renal fibrosis [6]. A study by Toki et al. [7] found a strong correlation between fibrosis and M2 macrophages observed in allogeneic renal transplant patients, with a higher infiltration of M2 macrophages being associated with a lower estimated glomerular filtration rate (eGFR). Similar findings were observed in a study by Ikezumi et al. [8], who found that infiltration of M2 macrophages was associated with an increase in interstitial fibrosis. Furthermore, a strong association between fibrotic areas and M2 macrophages was likewise found in the renal tissue of IgA nephropathy patients [9]. Thus, M2 macrophage infiltration plays a crucial role in renal fibrosis from various causes, and determining the mechanism of M2 macrophage infiltration is important to slow the progression of CKD in patients.

Recently, new disease-associated genes that can serve as diagnostic and prognostic biomarkers have been identified using microarray technology and comprehensive bioinformatics studies. However, the diagnostic role of genes associated with M2 macrophage infiltration in renal fibrosis remains uncertain. Therefore, in this study, the Gene Expression Omnibus (GEO) database provided us with a microarray dataset of uremic patients, and we used bioinformatics to identify biomarkers associated with M2 macrophage infiltration, while hoping to reveal the pathophysiological processes by which M2 macrophage infiltration promotes the development of renal fibrosis.

## 2. Materials and Methods

### 2.1. Methodological Type and Data Source

This research endeavor involved a bioinformatics investigation aimed at identifying datasets pertaining to renal fibrosis and uremia spanning the years 2010 to 2022. Ultimately, the GSE37171 dataset emerged as the most suitable candidate, encompassing a total of 40 control samples and 75 samples associated with uremia [10]. The GSE37171 dataset encompassed the acquisition of peripheral blood samples from individuals diagnosed with end-stage renal failure, as well as from healthy controls. These samples were subjected to genome-wide microarray analysis with the objective of investigating alterations in gene expression linked to uremia.

### 2.2. Disclosing Ethics Information

The protocol of this study was approved by the Institutional Review Board of the Second Affiliated Hospital of Nanchang University (Review [2022] 104). All study procedures involving human participants were in accordance with institutional and/or National Research Council ethical standards and the 1964 Helsinki Declaration.

### 2.3. Identification and Functional Enrichment Analysis of Differentially Expressed Genes (DEGs)

In the dataset GSE37171, the difference between the control and uremic samples was defined based on |log2FC| > 1 and adjusted for *p* < 0.05. It was identified using the Limma R package [11]. For the DEGs' pathway enrichment analysis, the ClusterProfiler R package [12] was used. As part of the Gene Ontology/Kyoto Encyclopedia of Genes and Genomes (GO/KEGG) analysis, BP, molecular functions (MF), and cellular components (CC) were included.

### 2.4. CIBERSORT

We used the CIBERSORT method for cell type identification to infer the abundance of different cell types, which was developed by Newman [13] et al. The CIBERSORT method is based on a known reference set that provides a gene expression signature set for 22 immune cell subtypes: leukocyte signature matrix (LM22). LM22 contains 547 genes and 22 immune cell types, including 7 T-cell types (follicular helper, regulatory [Tregs], gamma delta, CD8, CD4 naive, CD4 memory resting, and CD4 memory activated); naive and memory B-cells; active and resting dendritic cells; active and resting immune cells; resting and active macrophages; active mast cells and plasma cells; and eosinophils and neutrophils. *p* values, correlation coefficients, and root mean square errors were calculated for 22 immune cell subpopulations. Therefore, samples with CIBERSORT of *p* < 0.05 as the cutoff value and only samples with *p* < 0.05 were expected for further analysis. In addition, the number of permutations defining the feature matrix was 1000. Immune cells with expression of 0 were removed. Finally, 17 immune cells were selected for further analysis.

### 2.5. Weighted Gene Coexpression Network Analysis (WGCNA) Network Construction and Module Identification

We constructed coexpression networks using the WGCNA R package [14]. As a preliminary step, a clustering tree diagram was produced. Subsequently, gene expression profiles from the GSE37171 dataset and sample characteristics corresponding to different types of immune cells were used to calculate WGCNA. WGCNA uses a scale-free topology algorithm to calculate the soft threshold. This algorithm uses the power law distribution of the network to calculate the optimal soft threshold for the network. The soft threshold is then used to calculate the adjacency matrix, which is used to construct the network. We first transformed the adjacency matrix into a topological overlap matrix, and then used the degree of dissimilarity to establish the gene dendrogram and module color. By dynamic tree cutting, the original modules were further separated and combined with modules with similar functions. To determine which module (hub module) was most closely related to the sample traits, we calculated correlations between the module feature genes and sample traits. We selected the MElightgreen and MEgreen modules, which are most closely associated with M2 macrophages, based on the results of correlation analysis to identify hub genes.

### 2.6. Identification of Hub Gene and Construction of a Protein-Protein Interaction (PPI) Network

To analyze the interaction of M2 macrophage genes with possible proteins, a PPI network of M2 macrophage modules intersecting with DEGs was created using STRING 11.0 (https://string-db.org/) and Cytoscape (version 3.9.0) software [15]. To further understand the interactions between M2 macrophages and DEGs, the cytoHubba [16] plugin in Cytoscape software was used to identify closely linked gene center clusters. For further analysis, we selected clusters of 10 nodes only.

### 2.7. Identification of Biomarkers in M2 Macrophages

Least absolute shrinkage and selection operator (LASSO) [17] is used to scale down and select variables to reduce model complexity and improve predictive performance. Initially, genes from MElightgreen, MEgreen modules, and DEGs were subjected to WGCNA analysis to identify potential M2 macrophage infiltrating genes. Next, the LASSO method was used to select genes. We arbitrarily divided the GES37171 dataset into a training set (70%) and a testing set (30%) and analyzed the diagnostic power of the obtained genes on the training and testing sets using receiver operating characteristic (ROC) [18]. Subsequently, the selection and visualization of the most relevant features (SVM-RFE) method was implemented using the e1071 package [19, 20]. Potential genes were identified again by overlaying LASSO and SVM-RFE genes, and diagnostic accuracy was assessed using ROC curves.

### 2.8. Subject Characteristics and Quantitative Real-Time PCR (qPCR)

To determine whether the screened genes differ between healthy individuals and uremic patients, we obtained peripheral blood from healthy individuals and uremic patients for validation. Our study included individuals who were 18 years of age or older and diagnosed with CKD Stage 5 requiring dialysis treatment. However, patients with malignancy, acute infections such as pneumonia, acute heart failure, and severe anemia were excluded from the study. Secondly, we included individuals who exhibited normal liver and kidney function, serum lipids, blood pressure, blood glucose, and body mass index (BMI) levels, while also lacking risk factors for CKD such as hypertension, diabetes, and obesity.

Ten milliliter of blood was diluted 1:1 with phosphate-buffered saline (PBS) and 10 mL of Ficoll reagent (Solarbio, Beijing) and centrifuged at 400×*g* for 20 min at room temperature. Peripheral blood mononuclear cells at the interface of the PBS and Ficoll layers were collected in new tubes [21]. The resulting cells were washed three times with PBS, and the cell density was adjusted to 1 × 10^6^/mL using DMEM (Thermo Fisher Scientific, USA) containing 200 U/mL of double antibody. M2 macrophages were selected by flow sorting, inoculated into 24-well plates and cultured in an incubator at 37°C for 4 h. After 4 h, the supernatant was discarded and washed three times with PBS, and wall-adherent macrophages were obtained.

According to the manufacturer's directions, EASY spin Cellular RNA Rapid Extraction Kit (Aidlab Biotechnologies, Beijing) was utilized to extract RNA from the M2 macrophages of uremic patients (*N* = 10, 4 males and 6 females) and healthy individuals (*N* = 10, 4 males and 6 females). The Nanodrop2000 Nucleic Acid Protein Assay (Thermo Fisher Scientific, USA) was used to determine the concentration and purity of RNA. Reverse transcription of 1 *μ*g of total RNA per sample was performed using the SweScript RT I First Strand cDNA Synthesis Kit (Servicebio, Wuhan, China) according to the manufacturer's recommendations, followed by qPCR experiments. 2× SYBR Green qPCR Master Mix (Servicebio, Wuhan) was used for qPCR reactions, using 1 *μ*L of cDNA, and the desired number of individual primers in a total volume of 20 *μ*L. The experiments are performed in triplicate. Gene expression is calculated using the 2^−ΔΔCT^ method. Glyceraldehyde-3-phosphate dehydrogenase (GAPDH) is used as an internal reference gene. The primer sequences are as follows: adenomatous polyposis coli (APC)-F: AGCACAGCGAAGAATAGCCA, APC-R: TTGACCTTCATTCTGCCCGCT; zinc finger and BTB structural domain 2 (ZBTB2)-F: GGATTTGGCCAACCATGGAC, ZBTB2-R: TGGTTTCAAGCGGACACACT; and GAPDH-F: GTCAAGGCTGAGAACGGGAA, GAPDH-R: AAATGAGCCCCAGCCTTCTC.

### 2.9. Statistical Analysis

The DEGs for the dataset were acquired utilizing the Limma R package. Subsequently, the samples were subjected to immune infiltration analysis using the CIBERSORT method. Coexpression networks were then constructed using the WGCNA approach. Additionally, PPI networks were constructed, and hub genes were identified through the utilization of the STRING web page and Cytoscape software. Furthermore, the identification of overlapping genes associated with M2 macrophage infiltration was accomplished through the implementation of LASSO and SVM-RFE techniques. To compare data from two groups, the Wilcoxon test was used, and *p* < 0.05 was considered statistically significant. R was used to examine all of the data (version 4.2.0).

## 3. Results

### 3.1. Transcriptome Analysis of Uremic and Control Samples

**Figure 1:**
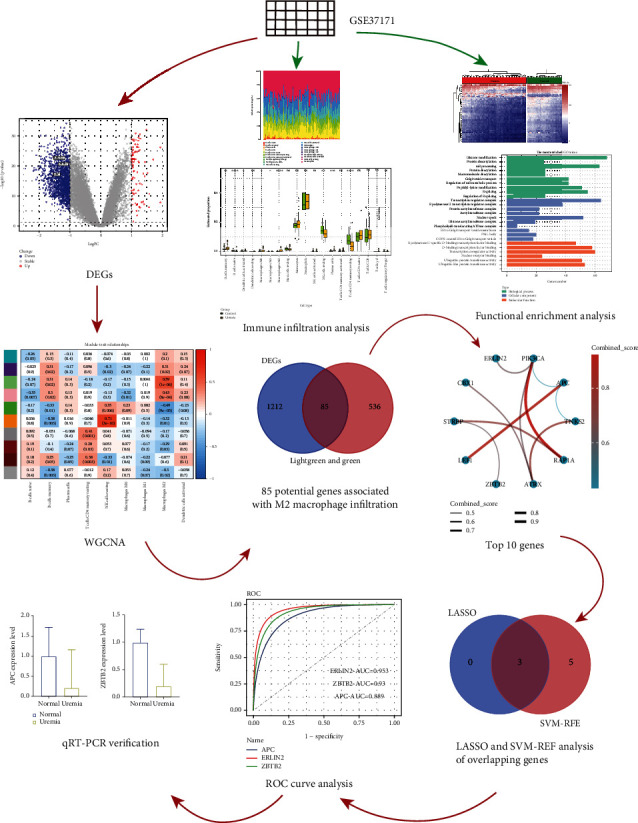
Workflow of this study.

**Figure 2:**
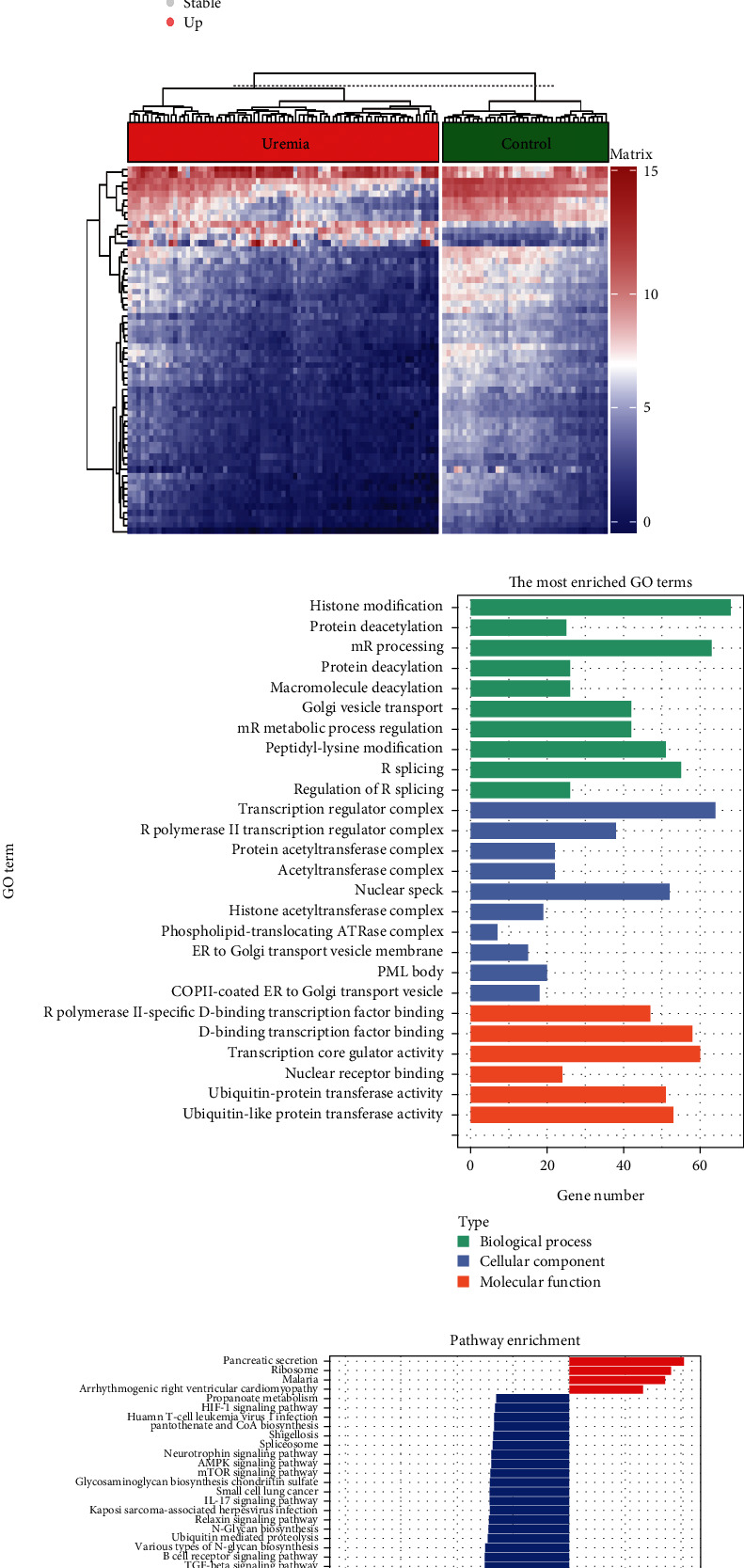
Analyses of the transcriptome profiles of both control and uremia samples. (a) Volcano plot showing DEGs between control and uremic groups. (b) Heat map showing 30 DEGs with the largest up or downregulation. (c) Bar graph showing the biological processes, cellular components, and molecular functions of DEGs enrichment. (d) Bar graph showing the KEGG pathway of DEGs enrichment.

The study flow is shown in Figure 1. The data preprocessing results are shown in Supporting Information 2, including principal component analysis and sample normalization processing. In the GSE37171 dataset, DEGs were observed to include 91 upregulated genes and 1207 downregulated genes compared to the control group (Figure 2(a)). Heat maps were used to show the expression of DEGs (Figure 2(b)). We analyzed the GO/KEGG pathways of DEGs to determine the biological functions of DEGs. Significant enrichment of DEGs was observed in 20 BP, 19 CC, 6 MF, and 70 KEGG pathways (Figures 2(c) and 2(d)). DEGs are mainly enriched in BP responsible for protein and mRNA processing, including histone modifications, protein deacetylation, mRNA processing, protein diacylation, macromolecular diacylation, Golgi vesicle transport, regulation of mRNA metabolic processes, peptidyl lysine modifications, and RNA splicing. DEGs were also heavily enriched in lymphocyte differentiation, and detailed results of the enrichment analysis can be found in Supporting Information 1. In addition to nuclear factor-kappa B (NF-*κ*B) signaling pathway, transforming growth factor-*β* (TGF-*β*) signaling pathway, mammalian target of rapamycin (mTOR) signaling pathway, phosphatidylinositol-3-kinase/protein kinase B (PI3K/Akt) signaling pathway, tumor necrosis factor (TNF) signaling pathway, Ras signaling pathway, and T cell receptor signaling pathway, DEGs were also found to be enriched in B cell receptor signaling pathway. Furthermore, a close association with uremia was shown in hepatitis B and C, *Yersinia pestis* infection, pathogenic *E. coli* infection, Epstein-Barr virus infection, human cytomegalovirus infection, *Salmonella* infection, measles, Chagas disease, leishmaniasis, and shigellosis (Supporting Information 1).

### 3.2. Immune Cell Infiltration in Uremia

**Figure 3:**
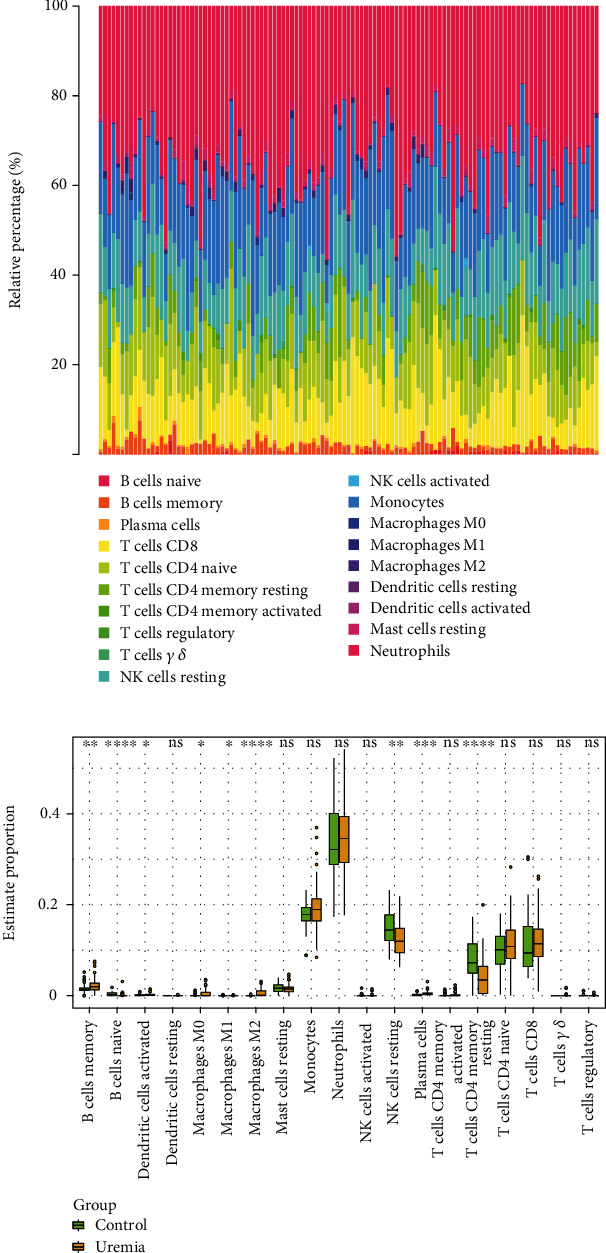
Landscape of the immune infiltration in control and uremia samples. (a) Heat map showing the immune infiltration in all samples analyzed by the CIBERSORT method. (b) Comparison of immune cell infiltration in control and uremic samples. ^∗^*p* < 0.05, ^∗∗^*p* < 0.01, ^∗∗∗^*p* < 0.001, ^∗∗∗∗^*p* < 0.0001, and ns = not significant.

We excluded immune cells with an abundance of 0. Therefore, only 17 immune cells were selected in the CIBERSORT study. Detailed results of the CIBERSORT analysis can be found in Supporting Information 1. Ratio histograms illustrate the differences in numbers between the different types of immune cells (Figure 3(a)). Nine immune cell types differed in number between the uremic and control groups (*p* < 0.05), with B-cell naive, macrophage M2, and T-cell CD4 memory type showing the most significant differences (*p* < 0.0001) (Figure 3(b)). There is growing evidence that uremia is associated with M2 macrophage infiltration [6, 22]. Overinfiltration of M2 macrophages may reveal the pathogenesis of progression of renal interstitial fibrosis. Therefore, we selected M2 macrophages for the next step of our study.

### 3.3. Obtained 85 Potential Genes Associated With M2 Macrophage Infiltration

**Figure 4:**
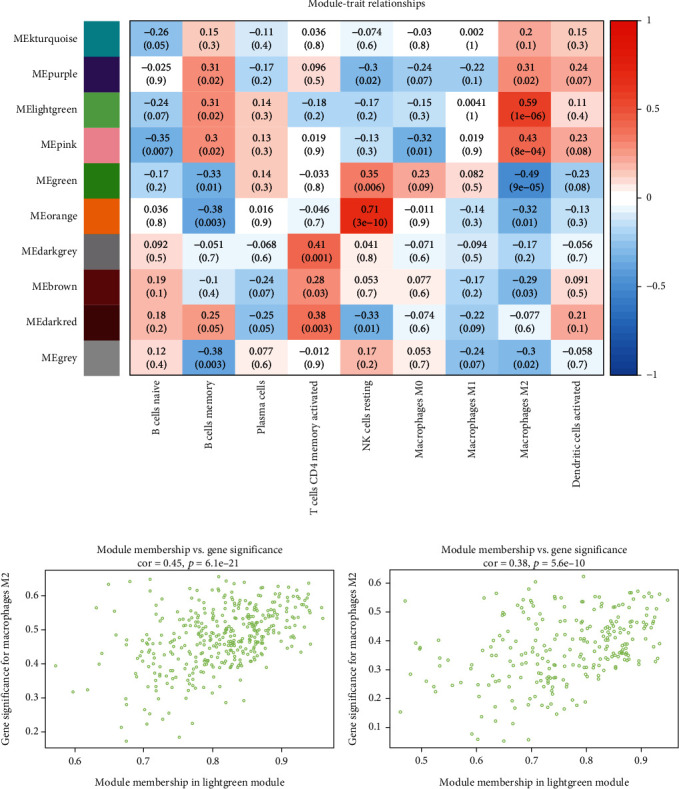
Identification related modules. (a) Evaluation of modules associated with immune infiltration in uremia. The correlation heat map demonstrates the correlation between modules and immune cells of different infiltrations. Each row represents a color-coded module and each column indicates one type of immune cell infiltrating into the tissue. The numbers for each cell represent the correlation coefficient and *p* value. (b) Scatter plot showing the relationship between the associated genes of M2 macrophages and the module members of MElightgreen. (c) Scatter plot showing the relationship between the associated genes of M2 macrophages and the module members of MEgreen.

**Figure 5:**
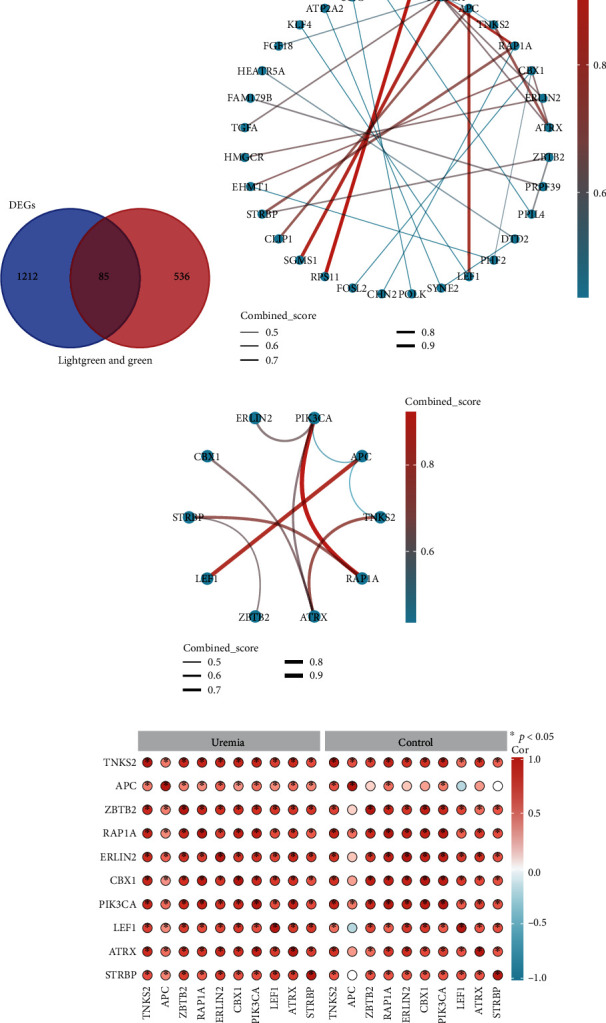
Identification of 10 hub genes. (a) Wayne diagram showing the 85 potential genes shared by DEGs and MElightgreen and MEgreen modules. (b) Cytoscape visualization showing the network diagram of protein-protein interactions. (c) Network diagram of hub gene junctions generated by cytoHubba plugin. (d) Correlation analysis of 10 pivotal genes between the two groups.

We identified modules related to M2 macrophages in uremia using the WGCNA method. After removing the outliers (Supporting Information 2), we created a sample dendrogram and a trait heat map (Supporting Information 2). The soft threshold is selected according to the function of the WGCNA, with an ideal soft threshold power of 18 and R^2^ of 0.85 (Supporting Information 2). After merging the comparable modules, the coexpression network showed 10 modules. Based on the module-trait relationships in Figure 4(a), we found that M2 macrophages were strongly correlated with MElightgreen (cor = 0.59, *p* < 0.05) and MEgreen (cor = −0.49, *p* < 0.05) modules. The characteristic genes of M2 macrophages showed a strong association with the modular genes in MElightgreen (cor = 0.45, *p* < 0.05) and MEgreen (cor = 0.38, *p* < 0.05) (Figures 4(b) and 4(c)). Therefore, for downstream analysis, we selected the MElightgreen and MEgreen modules. Next, we overlapped the DEGs with genes from the MElightgreen and MEgreen modules and identified 85 potential genes (Figure 5(a)).

### 3.4. Acquisition of Top 10 Genes

Next, based on the STRING database, we analyzed the PPI network of 85 candidate genes. In the web page, “multiple proteins” and “*Homo sapiens*” were selected. Network interaction links were considered statistically significant when the *p* value was less than 0.05, and interaction scores above 0.70 indicated a highly plausible relationship. A Cytoscape visualization of the PPI networks from the STRING database can be seen in Figure 5(b). Using the CytoHubba plugin, we identified the 10 most interlinked genes (Figure 5(c)). These 10 most interlinked genes include: ZBTB2, spermatid perinuclear RNA binding protein (STRBP), repressor activator protein 1A (RAP1A), lymphoid enhancer binding factor 1 (LEF1), APC, phosphatidylinositol-4,5-bisphosphate 3-kinase catalytic subunit alpha (PIK3CA), tankyrase 2 (TNKS2), ER lipid raft associated 2 (ERLIN2), chromatin remodeler (ATRX), and chromobox homolog 1 (CBX1). Then, as we can observe from Figure 5(d), APC and ZBTB2 correlate well with most of the genes in the uremia group. However, in the control group, APC correlated weakly with these genes.

### 3.5. Screening for ZBTB2, APC, and ERLIN2 Genes

**Figure 6:**
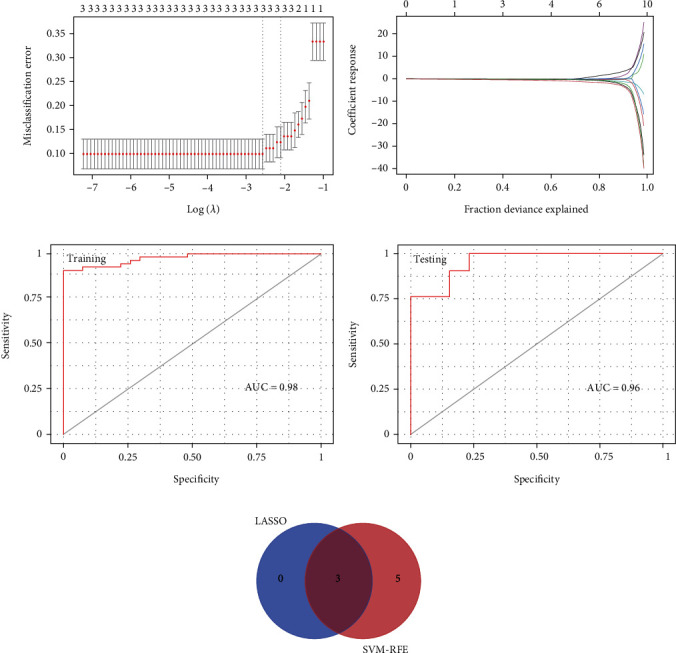
Identification of candidate genes by LASSO regression model and SVM-RFE. (a) Cross-validation to determine the best adjusted parameter log (lambda) for LASSO regression analysis. (b) LASSO coefficients of potential genes. (c, d) ROC curve analysis of the training and testing sets. (e) Venn diagram illustrating that there are three overlapping candidate genes in LASSO and SVM-RFE methods.

Subsequently, SVM-RFE and LASSO were used for identifying gene signatures from 10 candidate genes. The LASSO model (Figure 6(a)) was built by searching the hub gene expression profile. At lambda.1se = 0.009718, LASSO found nine gene features, which included ZBTB2, APC, and ERLIN2 (Figures 6(a) and 6(b)). The LASSO model allows the development of diagnostic markers for M2 macrophage infiltration and uremia. Using SVM-RFE, we identified eight gene signatures, including APC, TNKS2, RAP1A, ZBTB2, ERLIN2, CBX1, LEF1, and PIK3CA. For genes with stable expression in uremia, we overlapped the genes obtained by LASSO and SVM-RFE algorithms to obtain three genes (Figure 6(e)), including ZBTB2, APC, and ERLIN2. To validate the diagnostic performance of ZBTB2, APC, and ERLIN2, we divided the dataset into training and testing sets according to the scale. The results show that in the training set, the area under the curve (AUC) of the model is 0.98, while in the testing set, it is 0.96 (Figures 6(c) and 6(d)).

### 3.6. Validation of Potential Biomarkers of M2 Macrophage Infiltration in Uremic Samples

**Figure 7:**
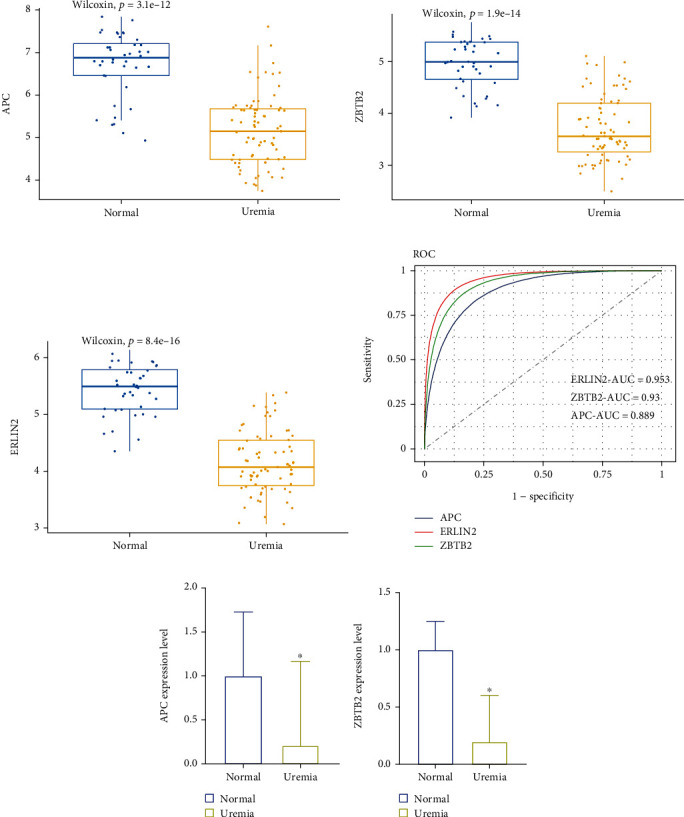
Validation of the obtained genes. (a–c) Expression levels of the three diagnostic biomarker candidate genes in the GSE37171 dataset in the control and uremic samples. (d) ROC curves to assess the accuracy of the three predictive biomarkers. 0: control samples; 1: uremic samples. (e, f) Expression levels of APC and ZBTB2 in human peripheral blood mononuclear cells were detected by qPCR. ^∗^*p* < 0.05.

In the GSE37171 dataset, ZBTB2, APC, and ERLIN2 were evaluated using ROC curves. We found that their expression levels were all significantly lower in the uremic samples than those in the control samples (Figure 7(a)–7(c)) and with high precision, with AUC of 0.889, 0.93, and 0.953, respectively (Figure 7(d)). These results suggest that ZBTB2, APC, and ERLIN2 can be used as diagnostic biomarkers of M2 macrophage infiltration in renal interstitial fibrosis. In addition, we performed external validation of these three genes. We found that the expression of APC and ZBTB2 was significantly lower in uremic patients than in healthy individuals. However, we found no difference in the expression of ERLIN2 in healthy individuals and uremic patients. In conclusion, these findings imply that APC and ZBTB2 may contribute to the progression of renal fibrosis by mediating M2 macrophage infiltration (Figures 7(e) and 7(f)).

## 4. Discussion

CKD is one of the leading causes of death in the world, and its incidence is steadily increasing worldwide [23]. Although scientists have conducted extensive studies on the mechanisms of renal fibrosis [24–26], treating renal fibrosis or preventing its progression remains a major challenge. Recent studies have shown that infiltration of M2 macrophages into the renal mesenchyme is essential for the development and progression of renal fibrosis [7]. Renal fibrosis is closely associated with infiltration of M2 macrophages [8, 9]. Since the mechanism of M2 macrophage infiltration in renal fibrosis is unknown. Therefore, a detailed study of the mechanisms of M2 macrophages in renal fibrosis is needed to provide new therapeutic strategies for patients with CKD. In this study, bioinformatics analysis revealed that uremic samples were enriched for several signaling pathways associated with uremic disease pathogenesis. In addition, the expression of APC and ZBTB2 was significantly decreased in M2 macrophages from uremic patients, and APC and ZBTB2 were identified as potential renal fibrosis biomarkers associated with M2 macrophage infiltration.

This study has identified multiple signaling pathways that are enriched in uremic samples, some of which are implicated in macrophage polarization and differentiation. Specifically, the NF-*κ*B signaling pathway has been recognized as a crucial pathway for macrophage activation and M1/M2 polarization [27, 28]. Inhibition of the NF-*κ*B signaling pathway has been shown to suppress M1 macrophage polarization and promote M2 macrophage polarization [29]. Similarly, activation of the mTOR signaling pathway and PI3K/Akt signaling pathway has been found to be essential for M2 macrophage polarization [30]. Moreover, the induction of M2 macrophage differentiation is facilitated by the TGF-*β* signaling pathway, which has been observed to be activated in M2 macrophages derived from injured kidneys [31, 32]. However, currently, there is a lack of literature documenting the correlation between M2 macrophages and the TNF signaling pathway, Ras signaling pathway, T cell receptor signaling pathway, and B cell receptor signaling pathway, although our bioinformatics analyses have revealed that these aforementioned signaling pathways are enriched in uremic samples.

M2 macrophages (also known as activated macrophages) are induced by IL-4. M2 macrophages express high levels of arginase-1 and dectin-1. This expression induces the production of proline in the nitric oxide metabolic pathway, which stimulates cell growth, collagen formation, and tissue repair [33]. Thus, M2 macrophages have a key role in tissue repair and fibrotic disease progression [34, 35]. It was found that in IgG4-related disease, M2 macrophages promote the production of several fibrogenic cytokines (IL-33, IL-1*β*, and TGF-*β*) via NF-*κ*B signaling, leading to severe fibrosis in the affected organs [36]. Furthermore, deletion of CCAAT/enhancer binding protein homologous protein promoted the expression of suppressor of cytokine signaling 1 and 3, which then inhibited signal transducer and activator of transcription 6/peroxisome proliferator-activated receptor gamma (STAT6/PPAR-*γ*) signaling, thereby attenuating the induction of M2 macrophages and alleviating idiopathic pulmonary fibrosis [37]. In contrast, IL-24 enhances STAT6/PPAR-*γ* signaling, thereby promoting IL-4-induced M2 macrophage production [38]. Importantly, there is growing evidence that M2 macrophage infiltration is associated with CKD [39, 40]. We found by bioinformatics analysis that in uremic samples, APC and ZBTB2 expressions were decreased in the infiltration module of M2 macrophages. Furthermore, we validated this in healthy individuals and uremic patients, and the results were consistent with the bioinformatics analysis. We hypothesize that APC and ZBTB2 are closely associated with M2 macrophage infiltration, and uncovering the role of APC and ABTB2 in M2 macrophage infiltration may unravel the physiopathological mechanisms underlying the development of renal fibrosis.

There is increasing evidence that infiltration of immune cells into the renal interstitial fluid leads to the development of renal fibrosis [41]. Bioinformatic investigations have also shown significant differences in the immune cell profile of healthy versus dysfunctional kidneys [42]. Our study found that uremia samples had increased abundances of B-cells memory, macrophages M0, macrophages M2, plasma cells, and activated dendritic cells, whereas T-cells CD4 naive, NK-cells activated, and B-cells naive were less invasive, indicating their crucial roles in the etiology of renal fibrosis. One study found a large number of M2 macrophages detected in renal tissue in a mouse model of ischemia-reperfusion [43]. In contrast, hydroxychloroquine reduces the infiltration of intrarenal macrophages, especially M2 macrophages, and reduces the degree of inflammation of tubulointerstitial fibrosis in vivo [44]. In addition, the number of infiltrating M2 macrophages was strongly correlated not only with the area of fibrosis but also with clinical examination indices [9]. There was a positive correlation between M2 macrophage infiltration and serum creatinine and 24-h proteinuria, but a negative correlation with eGFR [9]. As previously mentioned, extensive evidence and our current results suggest that M2 macrophages are key players in the development of renal fibrosis and should be the subject of further study, and mechanisms associated with M2 macrophage infiltration should be further explored.

APC is a large multidomain protein with a molecular mass of 300 kD [45]. Mutations in the APC gene are responsible for sporadic colorectal tumors and familial adenomatous polyposis [46, 47]. APC expression was positively correlated with arterial stiffness [44]. Furthermore, miR-142 acts as an inducer of fibrosis by targeting APC in cardiac fibroblasts [48]. Moreover, in a recent study on idiopathic membranous nephropathy (IMN) [49], APC expression was found to be significantly decreased in IMN patients. Unfortunately, there are no relevant studies to investigate the role of APC in renal fibrosis. APC was found to bind to *β*-catenin, a protein that plays a role in cell adhesion and Wnt signaling pathway [50]. APC is a negative regulator of the Wnt signaling pathway [51–53]. However, APC deletion leads to ligand nondependent pathway activation of the Wnt signaling pathway through lattice-protein-mediated endocytosis [54, 55]. Importantly, the Wnt signaling pathway is associated with M2 macrophage infiltration [56, 57]. Therefore, we hypothesized that the downregulation of APC could have mediated the infiltration of M2 macrophages through the activation of the Wnt signaling pathway, thereby promoting the progression of renal fibrosis.

ZBTB2 belongs to the POK family of transcription factors and has an n-terminal POZ structural domain and four c-terminal Krüppel-like C2H2 zinc fingers. The POZ structural domain is a conserved protein interaction pattern that frequently binds to transcriptional corepressors [58, 59]. ZBTB2 represses a variety of cellular promoters, including key regulators of the P53 DNA damage pathway [60]. ZBTB2 has been found to be associated with the development of a variety of tumors [61–65]. Unfortunately, there are no relevant studies to investigate the role of ZBTB2 in renal fibrosis. Interestingly, P53 and M2 macrophage polarization were closely related. Li et al. [66] found that P53 has a unique role in regulating M2 macrophage polarization. P53 acts as a physiological “brake” for M2 macrophage polarization through the murine double minute 2/cellular-myelocytomatosis viral oncogene axis. In addition, downregulation of P53 expression levels increases the polarization of M2 macrophages and regulates the tumor-associated microenvironment, ultimately leading to poor prognosis in lung cancer patients [67]. Therefore, we hypothesized that ZBTB2 may promote M2 macrophage infiltration through the P53 signaling pathway leading to the progression of renal fibrosis.

Our study showed that the expression of APC and ZBTB2 was closely correlated with the abundance of M2 macrophages, suggesting that APC and ZBTB2 may exacerbate renal fibrosis through infiltration of M2 macrophages. More critically, the levels of APC and ZBTB2 expressed by M2 macrophages were significantly lower in uremic patients compared with healthy subjects. Therefore, in delaying renal fibrosis, we speculate that APC and ZBTB2 may be potential therapeutic targets.

CKD is a complex disease that requires in-depth studies to determine its underlying mechanisms. Our study revealed that two genes, APC and ZBTB2, may be involved in the process of renal interstitial fibrosis. Further studies on the role of these two genes could provide valuable information to understand the pathogenesis of the disease. Specifically, these genes are thought to regulate the function of M2 macrophages, a type of immune cell involved in the development of renal interstitial fibrosis. Understanding how APC and ZBTB2 interact with M2 macrophages could pave the way for the development of new strategies for the treatment of CKD.

Several limitations of the current investigation must be acknowledged. First, the study is retrospective; in-depth studies are needed to clarify the role of APC and ZBTB2 in renal fibrosis. Second, we found only one suitable dataset. Our conclusions should be externally validated in additional datasets. Third, our results were only validated in M2 macrophages from uremic patients and healthy individuals, and further experiments in kidney tissues or cells are needed to confirm this result in the future. Additional in vitro and in vivo studies are necessary to investigate the specific mechanisms by which APC and ZBTB2 affect M2 macrophage infiltration in renal fibrosis. Notwithstanding the aforementioned limitations, it is crucial to acknowledge that our study has revealed a novel finding of diminished expression of APC and ZBTB2 in M2 macrophages among uremic patients. This reduced expression of APC and ZBTB2 in M2 macrophages serves as a significant indicator for the presence of renal fibrosis. Further investigations into the expression of APC and ZBTB2 in M2 macrophages hold promise for providing fresh insights into the mechanisms underlying renal fibrosis and facilitating the development of innovative therapeutic approaches to combat this disease.

## 5. Conclusion

Our findings identify biomarkers associated with M2 macrophage infiltration in renal fibrosis and speculate on possible mechanisms by which APC and ZBTB2 influence M2 macrophage infiltration to promote the progression of renal fibrosis, which may offer new possibilities to slow down the progression of the disease in patients with CKD.

## Data Availability

The datasets/analyses generated during the current study are available in the GEO database (https://www.ncbi.nlm.nih.gov/geo/query/acc.cgi?acc=GSE37171) and included in this published article.

## Nomenclature

APC: adenomatous polyposis coli
ATRX: chromatin remodeler
AUC: area under the curve
BP: biological processes
CBX1: chromobox homolog 1
CC: cellular components
CKD: chronic kidney disease
eGFR: estimated glomerular filtration rate
ERLIN2: ER lipid raft associated 2
GAPDH: glyceraldehyde-3-phosphate dehydrogenase
GEO: Gene Expression Omnibus
GO/KEGG: Gene Ontology/Kyoto Encyclopedia of Genes and Genomes
IMN: idiopathic membranous nephropathy
LASSO: least absolute shrinkage and selection operator
LEF1: lymphoid enhancer binding factor 1
MF: molecular functions
mTOR: mammalian target of rapamycin
PBS: phosphate-buffered saline
PI3K/Akt: phosphatidylinositol-3-kinase/protein kinase B
PIK3CA: phosphatidylinositol-4,5-bisphosphate 3-kinase catalytic subunit alpha
PPI: protein-protein interaction
qPCR: quantitative real-time PCR
RAP1A: repressor activator protein 1A
ROC: receiver operating characteristic
STAT6/PPAR-*γ*: signal transducer and activator of transcription 6/peroxisome proliferator-activated receptor gamma
STRBP: spermatid perinuclear RNA binding protein
SVM-RFE: selection and visualization of the most relevant features
TGF-*β*: transforming growth factor-*β*
TNF: tumor necrosis factor
TNKS2: tankyrase 2
WGCNA: weighted gene coexpression network analysis
ZBTB2: zinc finger and BTB structural domain 2

## Supporting Information

Supporting Information 1 Table S1: the result of the GO enrichment analysis. Table S2: the results of the KEGG enrichment pathway. Table S3: the result of immune infiltration for each sample.

Supporting Information 2 Figure S1: PCA and standardized analysis. (A) shows the PCA results of the two groups of samples, and (B) shows the histogram after sample normalization. Figure S2: sample clustering diagram. Figure S3: sample dendrogram and feature heat map demonstrating the correlation between samples and immune infiltrating cells. Figure S4: scale-free network test and choice of soft threshold. (A) is a scale-free network test, and (B) shows the choice of soft threshold.

## References

[1] Jager K. J., Kovesdy C., Langham R., Rosenberg M., Jha V., Zoccali C. (2019). A single number for advocacy and communication-worldwide more than 850 million individuals have kidney diseases. *Kidney International*.

[2] Foreman K. J., Marquez N., Dolgert A. (2018). Forecasting life expectancy, years of life lost, and all-cause and cause-specific mortality for 250 causes of death: reference and alternative scenarios for 2016-40 for 195 countries and territories. *Lancet*.

[3] Humphreys B. D. (2018). Mechanisms of renal fibrosis. *Annual Review of Physiology*.

[4] Tseng W. C., Tsai M. T., Chen N. J., Tarng D. C. (2020). Trichostatin A alleviates renal interstitial fibrosis through modulation of the M2 macrophage subpopulation. *International Journal of Molecular Sciences*.

[5] Ji X., Wang H., Wu Z. (2018). Specific inhibitor of Smad3 (SIS3) attenuates fibrosis, apoptosis, and inflammation in unilateral ureteral obstruction kidneys by inhibition of transforming growth factor *β* (TGF-*β*)/Smad3 signaling. *Medical Science Monitor*.

[6] Tang P. M., Nikolic-Paterson D. J., Lan H. Y. (2019). Macrophages: versatile players in renal inflammation and fibrosis. *Nature Reviews. Nephrology*.

[7] Toki D., Zhang W., Hor K. L. (2014). The role of macrophages in the development of human renal allograft fibrosis in the first year after transplantation. *American Journal of Transplantation*.

[8] Ikezumi Y., Suzuki T., Yamada T. (2015). Alternatively activated macrophages in the pathogenesis of chronic kidney allograft injury. *Pediatric Nephrology*.

[9] Yang M., Liu J. W., Zhang Y. T., Wu G. (2021). The role of renal macrophage, AIM, and TGF-*β*1 expression in renal fibrosis progression in IgAN patients. *Frontiers in Immunology*.

[10] Scherer A., Günther O. P., Balshaw R. F. (2013). Alteration of human blood cell transcriptome in uremia. *BMC Medical Genomics*.

[11] Ritchie M. E., Phipson B., Wu D. (2015). Limma powers differential expression analyses for RNA-sequencing and microarray studies. *Nucleic Acids Research*.

[12] Yu G., Wang L. G., Han Y., He Q. Y. (2012). clusterProfiler: an R package for comparing biological themes among gene clusters. *Omics: A Journal of Integrative Biology*.

[13] Newman A. M., Liu C. L., Green M. R. (2015). Robust enumeration of cell subsets from tissue expression profiles. *Nature Methods*.

[14] Langfelder P., Horvath S. (2008). WGCNA: an R package for weighted correlation network analysis. *BMC Bioinformatics*.

[15] Shannon P., Markiel A., Ozier O. (2003). Cytoscape: a software environment for integrated models of biomolecular interaction networks. *Genome Research*.

[16] Baranzini S. E., Madireddy L. R., Cromer A. (2015). Prognostic biomarkers of IFNb therapy in multiple sclerosis patients. *Multiple Sclerosis*.

[17] Vasquez M. M., Hu C., Roe D. J., Chen Z., Halonen M., Guerra S. (2016). Least absolute shrinkage and selection operator type methods for the identification of serum biomarkers of overweight and obesity: simulation and application. *BMC Medical Research Methodology*.

[18] Robin X., Turck N., Hainard A. (2011). pROC: an open-source package for R and S+ to analyze and compare ROC curves. *BMC Bioinformatics*.

[19] Guyon I., Weston J., Barnhill S., Vapnik V. (2002). Gene selection for cancer classification using support vector machines. *Machine Learning*.

[20] Noble W. S. (2006). What is a support vector machine?. *Nature Biotechnology*.

[21] Praekelt U., Kopp P. M., Rehm K. (2012). New isoform-specific monoclonal antibodies reveal different sub-cellular localisations for talin1 and talin2. *European Journal of Cell Biology*.

[22] Liu B., Jiang J., Liang H. (2021). Natural killer T cell/IL-4 signaling promotes bone marrow-derived fibroblast activation and M2 macrophage-to-myofibroblast transition in renal fibrosis. *International Immunopharmacology*.

[23] Rhee C. M., Kovesdy C. P. (2015). Epidemiology: spotlight on CKD deaths—increasing mortality worldwide. *Nature Reviews. Nephrology*.

[24] Geng X. Q., Ma A., He J. Z. (2020). Ganoderic acid hinders renal fibrosis via suppressing the TGF-*β*/Smad and MAPK signaling pathways. *Acta Pharmacologica Sinica*.

[25] Liu Y., Wang Y., Ding W., Wang Y. (2018). Mito-TEMPO alleviates renal fibrosis by reducing inflammation, mitochondrial dysfunction, and endoplasmic reticulum stress. *Oxidative Medicine and Cellular Longevity*.

[26] Li C., Shen Y., Huang L., Liu C., Wang J. (2021). Senolytic therapy ameliorates renal fibrosis postacute kidney injury by alleviating renal senescence. *The FASEB Journal*.

[27] Wanderley C. W., Colón D. F., Luiz J. P. M. (2018). Paclitaxel reduces tumor growth by reprogramming tumor-associated macrophages to an M1 profile in a TLR4-dependent manner. *Cancer Research*.

[28] Wang N., Liang H., Zen K. (2014). Molecular mechanisms that influence the macrophage M1–M2 polarization balance. *Frontiers in Immunology*.

[29] Liu C. P., Zhang X., Tan Q. L. (2017). NF-*κ*B pathways are involved in M1 polarization of RAW 264.7 macrophage by polyporus polysaccharide in the tumor microenvironment. *PLoS One*.

[30] Collins S. L., Oh M. H., Sun I. H. (2021). mTORC1 signaling regulates proinflammatory macrophage function and metabolism. *Journal of Immunology*.

[31] Feng Y., Liang Y., Zhu X. (2018). The signaling protein Wnt5a promotes TGF*β*1-mediated macrophage polarization and kidney fibrosis by inducing the transcriptional regulators Yap/Taz. *The Journal of Biological Chemistry*.

[32] Gratchev A. (2017). TGF-*β* signalling in tumour associated macrophages. *Immunobiology*.

[33] Hesse M., Modolell M., La Flamme A. C. (2001). Differential regulation of nitric oxide synthase-2 and arginase-1 by type 1/type 2 cytokines in vivo: granulomatous pathology is shaped by the pattern of L-arginine metabolism. *Journal of Immunology*.

[34] Sica A., Mantovani A. (2012). Macrophage plasticity and polarization: in vivo veritas. *The Journal of Clinical Investigation*.

[35] Venosa A., Malaviya R., Choi H., Gow A. J., Laskin J. D., Laskin D. L. (2016). Characterization of distinct macrophage subpopulations during nitrogen mustard-induced lung injury and fibrosis. *American Journal of Respiratory Cell and Molecular Biology*.

[36] Chinju A., Moriyama M., Kakizoe-Ishiguro N. (2022). CD163+ M2 macrophages promote fibrosis in IgG4-related disease via toll-like receptor 7/interleukin-1 receptor-associated kinase 4/NF-*κ*B signaling. *Arthritis & Rhematology*.

[37] Yao Y., Wang Y., Zhang Z. (2016). Chop deficiency protects mice against bleomycin-induced pulmonary fibrosis by attenuating M2 macrophage production. *Molecular Therapy*.

[38] Rao L. Z., Wang Y., Zhang L. (2021). IL-24 deficiency protects mice against bleomycin-induced pulmonary fibrosis by repressing IL-4-induced M2 program in macrophages. *Cell Death and Differentiation*.

[39] Chen J. F., Ni H. F., Pan M. M. (2013). Pirfenidone inhibits macrophage infiltration in 5/6 nephrectomized rats. *American Journal of Physiology. Renal Physiology*.

[40] Vielhauer V., Kulkarni O., Reichel C. A., Anders H. J. (2010). Targeting the recruitment of monocytes and macrophages in renal disease. *Seminars in Nephrology*.

[41] Chung K. W., Dhillon P., Huang S. (2019). Mitochondrial damage and activation of the STING pathway lead to renal inflammation and fibrosis. *Cell Metabolism*.

[42] Sun Y. C., Qiu Z. Z., Wen F. L., Yin J. Q., Zhou H. (2022). Revealing potential diagnostic gene biomarkers associated with immune infiltration in patients with renal fibrosis based on machine learning analysis. *Journal of Immunology Research*.

[43] Zheng H., Zhang Y., He J. (2021). Hydroxychloroquine inhibits macrophage activation and attenuates renal fibrosis after ischemia-reperfusion injury. *Frontiers in Immunology*.

[44] Kuipers A. L., Miljkovic I., Barinas-Mitchell E. (2020). Wnt pathway gene expression is associated with arterial stiffness. *Journal of the American Heart Association*.

[45] Groden J., Thliveris A., Samowitz W. (1991). Identification and characterization of the familial adenomatous polyposis coli gene. *Cell*.

[46] Nakamura Y. (1993). The role of the adenomatous polyposis coli (APC) gene in human cancers. *Advances in Cancer Research*.

[47] Kinzler K. W., Vogelstein B. (1996). Lessons from hereditary colorectal cancer. *Cell*.

[48] Cai L., Chao G., Li W. (2020). Activated CD4(+) T cells-derived exosomal miR-142-3p boosts post-ischemic ventricular remodeling by activating myofibroblast. *Aging (Albany NY)*.

[49] Ke B., Shen W., Liao Y., Hu J., Tu W., Fang X. (2023). APC ameliorates idiopathic membranous nephropathy by affecting podocyte apoptosis through the ERK1/2/YB-1/PLA2R1 axis. *Molecular and Cellular Biochemistry*.

[50] Rubinfeld B., Souza B., Albert I. (1993). Association of theAPCGene product with *β*-catenin. *Science*.

[51] Perrimon N. (1994). The genetic basis of patterned baldness in Drosophila. *Cell*.

[52] Miller J. R., Moon R. T. (1996). Signal transduction through beta-catenin and specification of cell fate during embryogenesis. *Genes & Development*.

[53] McGough I. J., Vincent J. P. (2018). APC moonlights to prevent Wnt signalosome assembly. *Developmental Cell*.

[54] Saito-Diaz K., Benchabane H., Tiwari A. (2018). APC inhibits ligand-independent Wnt signaling by the clathrin endocytic pathway. *Developmental Cell*.

[55] Bian J., Dannappel M., Wan C., Firestein R. (2020). Transcriptional regulation of Wnt/*β*-catenin pathway in colorectal cancer. *Cells*.

[56] van Ingen E., Foks A. C., Woudenberg T. (2021). Inhibition of microRNA-494-3p activates Wnt signaling and reduces proinflammatory macrophage polarization in atherosclerosis. *Molecular Therapy - Nucleic Acids*.

[57] Yang Y., Ye Y. C., Chen Y. (2018). Crosstalk between hepatic tumor cells and macrophages via Wnt/*β*-catenin signaling promotes M2-like macrophage polarization and reinforces tumor malignant behaviors. *Cell Death & Disease*.

[58] Siggs O. M., Beutler B. (2012). The BTB-ZF transcription factors. *Cell Cycle*.

[59] Lee S. U., Maeda T. (2012). POK/ZBTB proteins: an emerging family of proteins that regulate lymphoid development and function. *Immunological Reviews*.

[60] Kim M. Y., Koh D. I., Choi W. I. (2015). ZBTB2 increases PDK4 expression by transcriptional repression of RelA/p65. *Nucleic Acids Research*.

[61] Yang Y., Li H., He Z., Xie D., Ni J., Lin X. (2019). MicroRNA-488-3p inhibits proliferation and induces apoptosis by targeting ZBTB2 in esophageal squamous cell carcinoma. *Journal of Cellular Biochemistry*.

[62] Wang Y., Zheng X., Zhang Z. (2012). MicroRNA-149 inhibits proliferation and cell cycle progression through the targeting of ZBTB2 in human gastric cancer. *PLoS One*.

[63] Karemaker I. D., Vermeulen M. (2018). ZBTB2 reads unmethylated CpG island promoters and regulates embryonic stem cell differentiation. *EMBO Reports*.

[64] Du X., Wang J. M., Zhang D. L. (2021). AUF1 promotes proliferation and invasion of thyroid cancer via downregulation of ZBTB2 and subsequent TRIM58. *Frontiers in Oncology*.

[65] Zhi X. H., Jiang K., Ma Y. Y., Zhou L. Q. (2020). OIP5-AS1 promotes the progression of gastric cancer cells via the miR-153-3p/ZBTB2 axis. *European Review for Medical and Pharmacological Sciences*.

[66] Li L., Ng D. S., Mah W. C. (2015). A unique role for p53 in the regulation of M2 macrophage polarization. *Cell Death and Differentiation*.

[67] Chen Y. C., Young M. J., Chang H. P. (2022). Estradiol-mediated inhibition of DNMT1 decreases p53 expression to induce M2-macrophage polarization in lung cancer progression. *Oncogenesis*.

[68] Song J., Ke B., Fang X. (2023). APC and ZBTB2 may mediate M2 macrophage infiltration to promote the development of renal fibrosis: bioinformatics analysis. *no. PREPRINT (Version 1)*.

