# Supplementary material for: APC and ZBTB2 May Mediate M2 Macrophage Infiltration to Promote the Development of Renal Fibrosis: A Bioinformatics Analysis
Source: Biomed Res Int. 2024 Sep 18;2024:5674711. doi: 10.1155/2024/5674711 (PMC11424844; doi:10.1155/2024/5674711)
Supplement: Supporting Information 1 — Table S1: the result of the GO enrichment analysis. Table S2: the results of the KEGG enrichment pathway. Table S3: the result of immune infiltration for each sample. [file 5674711.f1.docx]

**Supplementary Table 1** the result of the GO enrichment analysis

| **ONTOLOGY** | **ID** | **Description** | **GeneRatio** | **BgRatio** | **pvalue** | **p.adjust** | **qvalue** | **Count** |
| --- | --- | --- | --- | --- | --- | --- | --- | --- |
| BP | GO:0016570 | histone modification | 68/1177 | 494/18800 | 6.86E-10 | 5.01E-06 | 4.57E-06 | 68 |
| BP | GO:0006476 | protein deacetylation | 25/1177 | 118/18800 | 6E-08 | 0.00017399 | 0.000158636 | 25 |
| BP | GO:0006397 | mRNA processing | 63/1177 | 500/18800 | 8.68E-08 | 0.00017399 | 0.000158636 | 63 |
| BP | GO:0035601 | protein deacylation | 26/1177 | 129/18800 | 9.53E-08 | 0.00017399 | 0.000158636 | 26 |
| BP | GO:0098732 | macromolecule deacylation | 26/1177 | 133/18800 | 1.81E-07 | 0.000263821 | 0.000240539 | 26 |
| BP | GO:0048193 | Golgi vesicle transport | 42/1177 | 294/18800 | 4.68E-07 | 0.000487646 | 0.000444612 | 42 |
| BP | GO:1903311 | regulation of mRNA metabolic process | 42/1177 | 294/18800 | 4.68E-07 | 0.000487646 | 0.000444612 | 42 |
| BP | GO:0018205 | peptidyl-lysine modification | 51/1177 | 392/18800 | 5.58E-07 | 0.000509573 | 0.000464604 | 51 |
| BP | GO:0008380 | RNA splicing | 55/1177 | 438/18800 | 6.4E-07 | 0.000518803 | 0.000473019 | 55 |
| BP | GO:0043484 | regulation of RNA splicing | 26/1177 | 154/18800 | 3.36E-06 | 0.002452395 | 0.002235976 | 26 |
| BP | GO:0000209 | protein polyubiquitination | 35/1177 | 244/18800 | 3.77E-06 | 0.002502752 | 0.002281889 | 35 |
| BP | GO:0043161 | proteasome-mediated ubiquitin-dependent protein catabolic process | 50/1177 | 414/18800 | 6.24E-06 | 0.00379631 | 0.003461293 | 50 |
| BP | GO:0097193 | intrinsic apoptotic signaling pathway | 39/1177 | 295/18800 | 8.02E-06 | 0.004505287 | 0.004107705 | 39 |
| BP | GO:0016575 | histone deacetylation | 19/1177 | 98/18800 | 8.99E-06 | 0.004689362 | 0.004275535 | 19 |
| BP | GO:0006473 | protein acetylation | 31/1177 | 216/18800 | 1.31E-05 | 0.006000522 | 0.005470988 | 31 |
| BP | GO:0043543 | protein acylation | 35/1177 | 258/18800 | 1.32E-05 | 0.006000522 | 0.005470988 | 35 |
| BP | GO:0043555 | regulation of translation in response to stress | 8/1177 | 20/18800 | 1.47E-05 | 0.006229135 | 0.005679426 | 8 |
| BP | GO:0006888 | endoplasmic reticulum to Golgi vesicle-mediated transport | 22/1177 | 129/18800 | 1.58E-05 | 0.006229135 | 0.005679426 | 22 |
| BP | GO:0018105 | peptidyl-serine phosphorylation | 40/1177 | 315/18800 | 1.62E-05 | 0.006229135 | 0.005679426 | 40 |
| BP | GO:0018209 | peptidyl-serine modification | 42/1177 | 338/18800 | 1.71E-05 | 0.006234483 | 0.005684302 | 42 |
| BP | GO:1903322 | positive regulation of protein modification by small protein conjugation or removal | 23/1177 | 140/18800 | 1.92E-05 | 0.006665293 | 0.006077094 | 23 |
| BP | GO:0007030 | Golgi organization | 25/1177 | 160/18800 | 2.06E-05 | 0.006847782 | 0.006243478 | 25 |
| BP | GO:0035459 | vesicle cargo loading | 9/1177 | 27/18800 | 0.000024 | 0.007617644 | 0.006945402 | 9 |
| BP | GO:1903320 | regulation of protein modification by small protein conjugation or removal | 33/1177 | 246/18800 | 2.89E-05 | 0.008779328 | 0.008004569 | 33 |
| BP | GO:0018394 | peptidyl-lysine acetylation | 27/1177 | 184/18800 | 3.12E-05 | 0.008779328 | 0.008004569 | 27 |
| BP | GO:0006417 | regulation of translation | 52/1177 | 463/18800 | 3.13E-05 | 0.008779328 | 0.008004569 | 52 |
| BP | GO:0030098 | lymphocyte differentiation | 45/1177 | 382/18800 | 3.33E-05 | 0.009005159 | 0.008210472 | 45 |
| CC | GO:0005667 | transcription regulator complex | 64/1222 | 483/19594 | 8.83E-09 | 7.84E-06 | 6.87E-06 | 64 |
| CC | GO:0090575 | RNA polymerase II transcription regulator complex | 38/1222 | 230/19594 | 3.34E-08 | 1.48E-05 | 1.30E-05 | 38 |
| CC | GO:0031248 | protein acetyltransferase complex | 22/1222 | 97/19594 | 9.71E-08 | 2.15E-05 | 1.89E-05 | 22 |
| CC | GO:1902493 | acetyltransferase complex | 22/1222 | 97/19594 | 9.71E-08 | 2.15E-05 | 1.89E-05 | 22 |
| CC | GO:0016607 | nuclear speck | 52/1222 | 411/19594 | 9.26E-07 | 0.000164484 | 0.000144089 | 52 |
| CC | GO:0000123 | histone acetyltransferase complex | 19/1222 | 87/19594 | 1.33E-06 | 0.000196148 | 0.000171827 | 19 |
| CC | GO:1990531 | phospholipid-translocating ATPase complex | 7/1222 | 13/19594 | 4.44E-06 | 0.000494627 | 0.000433297 | 7 |
| CC | GO:0012507 | ER to Golgi transport vesicle membrane | 15/1222 | 62/19594 | 4.46E-06 | 0.000494627 | 0.000433297 | 15 |
| CC | GO:0016605 | PML body | 20/1222 | 105/19594 | 6.61E-06 | 0.000651853 | 0.000571028 | 20 |
| CC | GO:0030134 | COPII-coated ER to Golgi transport vesicle | 18/1222 | 94/19594 | 1.75E-05 | 0.001429107 | 0.001251909 | 18 |
| CC | GO:0005637 | nuclear inner membrane | 14/1222 | 61/19594 | 1.77E-05 | 0.001429107 | 0.001251909 | 14 |
| CC | GO:0098687 | chromosomal region | 43/1222 | 366/19594 | 4.85E-05 | 0.003591863 | 0.003146499 | 43 |
| CC | GO:0031228 | intrinsic component of Golgi membrane | 14/1222 | 67/19594 | 5.42E-05 | 0.003701271 | 0.003242341 | 14 |
| CC | GO:0030173 | integral component of Golgi membrane | 13/1222 | 62/19594 | 9.57E-05 | 0.006067658 | 0.005315315 | 13 |
| CC | GO:0031965 | nuclear membrane | 36/1222 | 300/19594 | 0.000127 | 0.007512207 | 0.00658075 | 36 |
| CC | GO:0070971 | endoplasmic reticulum exit site | 9/1222 | 33/19594 | 0.000136 | 0.007574963 | 0.006635725 | 9 |
| CC | GO:0030127 | COPII vesicle coat | 6/1222 | 15/19594 | 0.000178 | 0.009235306 | 0.008090198 | 6 |
| CC | GO:0035770 | ribonucleoprotein granule | 32/1222 | 261/19594 | 0.000199 | 0.009235306 | 0.008090198 | 32 |
| CC | GO:0008287 | protein serine/threonine phosphatase complex | 11/1222 | 50/19594 | 0.000208 | 0.009235306 | 0.008090198 | 11 |
| CC | GO:1903293 | phosphatase complex | 11/1222 | 50/19594 | 0.000208 | 0.009235306 | 0.008090198 | 11 |
| MF | GO:0061629 | RNA polymerase II-specific DNA-binding transcription factor binding | 47/1208 | 348/18410 | 1.94E-06 | 0.001671802 | 0.001548713 | 47 |
| MF | GO:0140297 | DNA-binding transcription factor binding | 58/1208 | 470/18410 | 2.51E-06 | 0.001671802 | 0.001548713 | 58 |
| MF | GO:0003712 | transcription coregulator activity | 60/1208 | 497/18410 | 3.44E-06 | 0.001671802 | 0.001548713 | 60 |
| MF | GO:0016922 | nuclear receptor binding | 24/1208 | 139/18410 | 1.17E-05 | 0.00425081 | 0.003937838 | 24 |
| MF | GO:0004842 | ubiquitin-protein transferase activity | 51/1208 | 433/18410 | 3.56E-05 | 0.00990796 | 0.009178471 | 51 |
| MF | GO:0019787 | ubiquitin-like protein transferase activity | 53/1208 | 458/18410 | 4.07E-05 | 0.00990796 | 0.009178471 | 53 |

**Supplementary Table 2** the results of the KEGG enrichment pathway.

| **ID** | **Description** | **GeneRatio** | **BgRatio** | **pvalue** | **p.adjust** | **qvalue** | **Count** |
| --- | --- | --- | --- | --- | --- | --- | --- |
| hsa05210 | Colorectal cancer | 16/494 | 86/8157 | 4.78E-05 | 0.008067098 | 0.00656175 | 16 |
| hsa05211 | Renal cell carcinoma | 14/494 | 69/8157 | 5.24E-05 | 0.008067098 | 0.00656175 | 14 |
| hsa05161 | Hepatitis B | 23/494 | 162/8157 | 0.000107 | 0.009428895 | 0.00766944 | 23 |
| hsa04660 | T cell receptor signaling pathway | 17/494 | 104/8157 | 0.000151 | 0.009428895 | 0.00766944 | 17 |
| hsa04012 | ErbB signaling pathway | 15/494 | 85/8157 | 0.000153 | 0.009428895 | 0.00766944 | 15 |
| hsa05135 | Yersinia infection | 20/494 | 137/8157 | 0.000203 | 0.010444913 | 0.00849586 | 20 |
| hsa04141 | Protein processing in endoplasmic reticulum | 23/494 | 171/8157 | 0.000243 | 0.010712876 | 0.00871382 | 23 |
| hsa05130 | Pathogenic Escherichia coli infection | 24/494 | 197/8157 | 0.000788 | 0.029939444 | 0.02435266 | 24 |
| hsa04068 | FoxO signaling pathway | 18/494 | 131/8157 | 0.000875 | 0.029939444 | 0.02435266 | 18 |
| hsa04110 | Cell cycle | 17/494 | 126/8157 | 0.00148 | 0.045574599 | 0.03707025 | 17 |
| hsa05212 | Pancreatic cancer | 12/494 | 76/8157 | 0.00189 | 0.05293008 | 0.04305318 | 12 |
| hsa01521 | EGFR tyrosine kinase inhibitor resistance | 12/494 | 79/8157 | 0.002646 | 0.067925902 | 0.05525073 | 12 |
| hsa04014 | Ras signaling pathway | 25/494 | 235/8157 | 0.004122 | 0.081779055 | 0.06651885 | 25 |
| hsa04115 | p53 signaling pathway | 11/494 | 73/8157 | 0.004188 | 0.081779055 | 0.06651885 | 11 |
| hsa05162 | Measles | 17/494 | 139/8157 | 0.00424 | 0.081779055 | 0.06651885 | 17 |
| hsa03250 | Viral life cycle - HIV-1 | 10/494 | 63/8157 | 0.004248 | 0.081779055 | 0.06651885 | 10 |
| hsa04140 | Autophagy - animal | 17/494 | 141/8157 | 0.004908 | 0.083981715 | 0.06831049 | 17 |
| hsa04659 | Th17 cell differentiation | 14/494 | 108/8157 | 0.005415 | 0.083981715 | 0.06831049 | 14 |
| hsa05417 | Lipid and atherosclerosis | 23/494 | 215/8157 | 0.005428 | 0.083981715 | 0.06831049 | 23 |
| hsa03015 | mRNA surveillance pathway | 13/494 | 97/8157 | 0.005454 | 0.083981715 | 0.06831049 | 13 |
| hsa05220 | Chronic myeloid leukemia | 11/494 | 76/8157 | 0.005726 | 0.083981715 | 0.06831049 | 11 |
| hsa05225 | Hepatocellular carcinoma | 19/494 | 168/8157 | 0.00613 | 0.085819451 | 0.0698053 | 19 |
| hsa05235 | PD-L1 expression and PD-1 checkpoint pathway in cancer | 12/494 | 89/8157 | 0.007096 | 0.095022534 | 0.07729106 | 12 |
| hsa04668 | TNF signaling pathway | 14/494 | 112/8157 | 0.007467 | 0.095829163 | 0.07794717 | 14 |
| hsa00020 | Citrate cycle (TCA cycle) | 6/494 | 30/8157 | 0.008175 | 0.096640471 | 0.07860708 | 6 |
| hsa05142 | Chagas disease | 13/494 | 102/8157 | 0.008318 | 0.096640471 | 0.07860708 | 13 |
| hsa04151 | PI3K-Akt signaling pathway | 33/494 | 354/8157 | 0.008472 | 0.096640471 | 0.07860708 | 33 |
| hsa04710 | Circadian rhythm | 6/494 | 31/8157 | 0.009631 | 0.105941273 | 0.08617233 | 6 |
| hsa04936 | Alcoholic liver disease | 16/494 | 142/8157 | 0.011761 | 0.121283304 | 0.09865149 | 16 |
| hsa04071 | Sphingolipid signaling pathway | 14/494 | 119/8157 | 0.012504 | 0.121283304 | 0.09865149 | 14 |
| hsa04114 | Oocyte meiosis | 15/494 | 131/8157 | 0.012585 | 0.121283304 | 0.09865149 | 15 |
| hsa04218 | Cellular senescence | 17/494 | 156/8157 | 0.013134 | 0.121283304 | 0.09865149 | 17 |
| hsa04152 | AMPK signaling pathway | 14/494 | 120/8157 | 0.013399 | 0.121283304 | 0.09865149 | 14 |
| hsa04070 | Phosphatidylinositol signaling system | 12/494 | 97/8157 | 0.013781 | 0.121283304 | 0.09865149 | 12 |
| hsa05160 | Hepatitis C | 17/494 | 157/8157 | 0.013934 | 0.121283304 | 0.09865149 | 17 |
| hsa04066 | HIF-1 signaling pathway | 13/494 | 109/8157 | 0.014176 | 0.121283304 | 0.09865149 | 13 |
| hsa05231 | Choline metabolism in cancer | 12/494 | 98/8157 | 0.014874 | 0.122918362 | 0.09998144 | 12 |
| hsa05166 | Human T-cell leukemia virus 1 infection | 22/494 | 222/8157 | 0.015165 | 0.122918362 | 0.09998144 | 22 |
| hsa05170 | Human immunodeficiency virus 1 infection | 21/494 | 212/8157 | 0.017488 | 0.138106553 | 0.11233547 | 21 |
| hsa04211 | Longevity regulating pathway | 11/494 | 89/8157 | 0.018066 | 0.138129479 | 0.11235412 | 11 |
| hsa04910 | Insulin signaling pathway | 15/494 | 137/8157 | 0.018387 | 0.138129479 | 0.11235412 | 15 |
| hsa05169 | Epstein-Barr virus infection | 20/494 | 202/8157 | 0.020175 | 0.147423267 | 0.11991366 | 20 |
| hsa05134 | Legionellosis | 8/494 | 57/8157 | 0.020582 | 0.147423267 | 0.11991366 | 8 |
| hsa05222 | Small cell lung cancer | 11/494 | 92/8157 | 0.022625 | 0.15647292 | 0.12727462 | 11 |
| hsa04064 | NF-kappa B signaling pathway | 12/494 | 104/8157 | 0.022861 | 0.15647292 | 0.12727462 | 12 |
| hsa04926 | Relaxin signaling pathway | 14/494 | 129/8157 | 0.023849 | 0.157099896 | 0.1277846 | 14 |
| hsa04120 | Ubiquitin mediated proteolysis | 15/494 | 142/8157 | 0.024624 | 0.157099896 | 0.1277846 | 15 |
| hsa05167 | Kaposi sarcoma-associated herpesvirus infection | 19/494 | 194/8157 | 0.025623 | 0.157099896 | 0.1277846 | 19 |
| hsa04662 | B cell receptor signaling pathway | 10/494 | 82/8157 | 0.025635 | 0.157099896 | 0.1277846 | 10 |
| hsa04350 | TGF-beta signaling pathway | 11/494 | 94/8157 | 0.026099 | 0.157099896 | 0.1277846 | 11 |
| hsa04657 | IL-17 signaling pathway | 11/494 | 94/8157 | 0.026099 | 0.157099896 | 0.1277846 | 11 |
| hsa04150 | mTOR signaling pathway | 16/494 | 156/8157 | 0.026523 | 0.157099896 | 0.1277846 | 16 |
| hsa04722 | Neurotrophin signaling pathway | 13/494 | 119/8157 | 0.027462 | 0.15958885 | 0.12980911 | 13 |
| hsa00513 | Various types of N-glycan biosynthesis | 6/494 | 39/8157 | 0.028417 | 0.160953759 | 0.13091933 | 6 |
| hsa00532 | Glycosaminoglycan biosynthesis - chondroitin sulfate / dermatan sulfate | 4/494 | 20/8157 | 0.029667 | 0.160953759 | 0.13091933 | 4 |
| hsa03013 | Nucleocytoplasmic transport | 12/494 | 108/8157 | 0.02971 | 0.160953759 | 0.13091933 | 12 |
| hsa00510 | N-Glycan biosynthesis | 7/494 | 50/8157 | 0.029787 | 0.160953759 | 0.13091933 | 7 |
| hsa04919 | Thyroid hormone signaling pathway | 13/494 | 121/8157 | 0.030955 | 0.163766666 | 0.13320734 | 13 |
| hsa05163 | Human cytomegalovirus infection | 21/494 | 225/8157 | 0.031392 | 0.163766666 | 0.13320734 | 21 |
| hsa05215 | Prostate cancer | 11/494 | 97/8157 | 0.032011 | 0.163766666 | 0.13320734 | 11 |
| hsa04213 | Longevity regulating pathway - multiple species | 8/494 | 62/8157 | 0.032434 | 0.163766666 | 0.13320734 | 8 |
| hsa00770 | Pantothenate and CoA biosynthesis | 4/494 | 21/8157 | 0.034948 | 0.173610494 | 0.14121428 | 4 |
| hsa05226 | Gastric cancer | 15/494 | 149/8157 | 0.035849 | 0.17526223 | 0.14255779 | 15 |
| hsa00640 | Propanoate metabolism | 5/494 | 32/8157 | 0.041473 | 0.19666625 | 0.15996776 | 5 |
| hsa04371 | Apelin signaling pathway | 14/494 | 139/8157 | 0.041528 | 0.19666625 | 0.15996776 | 14 |
| hsa05140 | Leishmaniasis | 9/494 | 77/8157 | 0.042143 | 0.19666625 | 0.15996776 | 9 |
| hsa05131 | Shigellosis | 22/494 | 247/8157 | 0.04388 | 0.201718464 | 0.16407722 | 22 |
| hsa04380 | Osteoclast differentiation | 13/494 | 128/8157 | 0.045718 | 0.207074149 | 0.16843352 | 13 |
| hsa05132 | Salmonella infection | 22/494 | 249/8157 | 0.047253 | 0.210925439 | 0.17156615 | 22 |
| hsa03018 | RNA degradation | 9/494 | 79/8157 | 0.048412 | 0.213010726 | 0.17326231 | 9 |
|  |  |  |  |  |  |  |  |

**Supplementary Table 3** the result of immune infiltration for each sample.

|  | **B cells naive** | **B cells memory** | **Plasma cells** | **T cells CD8** | **T cells CD4 naive** | **T cells CD4 memory resting** | **T cells CD4 memory activated** | **T cells regulatory (Tregs)** | **T cells gamma delta** | **NK cells resting** | **NK cells activated** | **Monocytes** | **Macrophages M0** | **Macrophages M1** | **Macrophages M2** | **Dendritic cells resting** | **Dendritic cells activated** | **Mast cells resting** | **Neutrophils** |
| --- | --- | --- | --- | --- | --- | --- | --- | --- | --- | --- | --- | --- | --- | --- | --- | --- | --- | --- | --- |
| GSM912761 | 0 | 0.00560528 | 0.0056927 | 0.182321866 | 0.140152654 | 0.02588975 | 0.000481675 | 0 | 0 | 0.175776347 | 0 | 0.20546416 | 0 | 0 | 0 | 0 | 8.05E-05 | 0.014598755 | 0.243936313 |
| GSM912762 | 0 | 0.029051535 | 0.00490052 | 0.138742355 | 0.170078751 | 0.00926481 | 0 | 0 | 0 | 0.110698202 | 0 | 0.15246153 | 0 | 0 | 0.017019673 | 0 | 0.000367486 | 0.025409546 | 0.342005589 |
| GSM912763 | 0 | 0.014574083 | 0.00368347 | 0.036167877 | 0.104549611 | 0.078065193 | 0 | 0 | 0 | 0.131958426 | 0 | 0.16379948 | 0.002214072 | 0 | 0.001878226 | 0 | 0 | 0.000375377 | 0.462734194 |
| GSM912764 | 0 | 0.071378651 | 0.0145717 | 0.164050122 | 0.078437065 | 0.034538296 | 0.007518869 | 0 | 0 | 0.123066134 | 0 | 0.24302352 | 0 | 0 | 0.003645944 | 0 | 0.00193866 | 0.000142139 | 0.257688894 |
| GSM912765 | 0 | 0.013215911 | 0.00643653 | 0.262571829 | 0.01395719 | 0 | 0 | 0 | 0 | 0.183953175 | 0 | 0.15895807 | 0.009691727 | 0 | 0 | 0 | 0 | 0.019005172 | 0.332210397 |
| GSM912766 | 0 | 0.010038288 | 0.0077386 | 0.115330319 | 0.089628993 | 0.026207556 | 0 | 0 | 0 | 0.131212909 | 0 | 0.20015534 | 0.009208676 | 0 | 0.020114772 | 0 | 0.002642538 | 0.012203621 | 0.375518386 |
| GSM912767 | 0 | 0.032228715 | 0.00678303 | 0.016187307 | 0.17394497 | 0.018272474 | 0 | 0 | 0 | 0.125289089 | 0 | 0.25434735 | 0.035477316 | 0 | 0 | 0 | 0.009651451 | 0.004606906 | 0.32321139 |
| GSM912768 | 0 | 0.050898997 | 0 | 0.080658583 | 0.135318491 | 0 | 0 | 0 | 0 | 0.109075185 | 0 | 0.19019574 | 0.015699603 | 0.001934641 | 0.031290723 | 0 | 0 | 0.035658508 | 0.349269532 |
| GSM912769 | 0 | 0.037641987 | 0.00863416 | 0.126712165 | 0.172187298 | 0.073989511 | 0 | 0.002097516 | 0 | 0.101273309 | 0 | 0.14037966 | 0.006633112 | 0 | 0 | 0 | 0.001640319 | 0.011903666 | 0.316907299 |
| GSM912770 | 0 | 0.075535314 | 0.03083886 | 0.126359146 | 0.185103801 | 0.009213926 | 0.003592671 | 0 | 0 | 0.111916578 | 0 | 0.20412618 | 0 | 0 | 0.002713572 | 0 | 0.002113274 | 0.00185518 | 0.246631503 |
| GSM912771 | 0 | 0.034134599 | 0.0013777 | 0.128333984 | 0.107971904 | 0 | 0.00225351 | 0 | 0 | 0.075683111 | 0 | 0.16864812 | 0.001466656 | 0 | 0 | 0 | 0.001309466 | 0.01073607 | 0.468084878 |
| GSM912772 | 0 | 0.012272141 | 0.00257118 | 0.119312987 | 0.149083598 | 0.09019962 | 0 | 0 | 0 | 0.119667247 | 0 | 0.21566061 | 0 | 0 | 0 | 0 | 0.001263101 | 0.012747939 | 0.27722158 |
| GSM912773 | 0 | 0.022301016 | 0.00468379 | 0.102805475 | 0.220182326 | 0.096873262 | 0 | 0 | 0 | 0.157860088 | 0 | 0.16045064 | 0 | 0 | 0 | 0 | 0 | 0.005071711 | 0.229771699 |
| GSM912774 | 0.005842315 | 0.012167997 | 0.00162995 | 0.147684369 | 0.098949333 | 0.059233571 | 0 | 0.001946317 | 0 | 0.162613988 | 0 | 0.19991031 | 0.008206678 | 0 | 0 | 0 | 0.000550035 | 0.007348834 | 0.2939163 |
| GSM912775 | 0 | 0.038116069 | 0.00391954 | 0.173537277 | 0.085583116 | 0 | 0 | 0 | 0 | 0.211899852 | 0 | 0.10184063 | 0 | 0 | 0.004739535 | 0 | 0.002966505 | 0.002776009 | 0.374621462 |
| GSM912776 | 0 | 0.021246528 | 0.00378024 | 0.051129628 | 0.164684148 | 0.011206705 | 0 | 0 | 0 | 0.09027571 | 0 | 0.10592861 | 0 | 0 | 0.007907412 | 0 | 0.004058885 | 0.011837806 | 0.527944327 |
| GSM912777 | 0.031158775 | 0.012976741 | 0 | 0.085936044 | 0.160998371 | 0.109991808 | 0 | 0 | 0 | 0.098400826 | 0 | 0.20177445 | 0.004329423 | 0 | 0 | 0 | 0 | 0 | 0.294433566 |
| GSM912778 | 0 | 0.064923639 | 0.01062926 | 0.113542758 | 0.124843369 | 0.050402589 | 0 | 0 | 0 | 0.108647321 | 0 | 0.18681845 | 0 | 0 | 0 | 0 | 0.001921102 | 0.018031641 | 0.320239877 |
| GSM912779 | 0 | 0.017750726 | 0.00345261 | 0.115254544 | 0.104911653 | 0.030766116 | 0 | 0 | 0 | 0.147268267 | 0 | 0.18476827 | 0 | 0 | 0 | 0 | 0.001969779 | 0 | 0.393858036 |
| GSM912780 | 0 | 0.017620511 | 0.00297937 | 0.096489606 | 0.059819949 | 0.035496867 | 0.016847568 | 0 | 0 | 0.20709288 | 0 | 0.16492835 | 0 | 0 | 0.007408024 | 0.001212219 | 0.00274872 | 0.042600586 | 0.344755356 |
| GSM912781 | 0 | 0 | 0.01683075 | 0.116204607 | 0.074559574 | 0 | 0 | 0 | 0 | 0.068171759 | 0 | 0.2723731 | 0.006162763 | 0 | 0 | 0 | 0.003579146 | 0.034761352 | 0.40735695 |
| GSM912782 | 0 | 0.021332922 | 0.00316987 | 0.097680934 | 0.123399399 | 0.024958076 | 0.00071564 | 0 | 0 | 0.089018162 | 0 | 0.17130466 | 0 | 0 | 0.020277965 | 0 | 0.003069583 | 0.012181126 | 0.432891665 |
| GSM912783 | 0 | 0.025027028 | 0.00095272 | 0.124811354 | 0.17810774 | 0.056803902 | 0.006849609 | 0 | 0.004005252 | 0.079718386 | 0 | 0.18081718 | 0 | 0 | 0.021582651 | 0.002413007 | 0.009029053 | 0.021362633 | 0.288519478 |
| GSM912784 | 0 | 0.023708756 | 0 | 0.009386427 | 0.107968144 | 0.06690186 | 0.01591957 | 0 | 0 | 0.072255811 | 0 | 0.16126844 | 0 | 0 | 0 | 0 | 0.002866098 | 0.024136743 | 0.515588152 |
| GSM912785 | 0 | 0.031046683 | 0.00223042 | 0.157603015 | 0.106690929 | 0.025124299 | 0 | 0 | 0 | 0.112619227 | 0 | 0.1964942 | 0 | 0 | 0.023411623 | 0 | 0.0031491 | 0.023265088 | 0.318365413 |
| GSM912786 | 0 | 0.023242552 | 0.00079025 | 0.134550079 | 0.127308686 | 0 | 0.005238822 | 0 | 0 | 0.092984081 | 0 | 0.1873745 | 0 | 0.000295234 | 0.023400581 | 0 | 0.014764839 | 0.017505559 | 0.372544813 |
| GSM912787 | 0 | 0.046938357 | 0 | 0.051270394 | 0.139428793 | 0.04181806 | 0.00305459 | 0 | 0 | 0.073101612 | 0 | 0.20963461 | 0 | 0 | 0 | 0 | 0.002606988 | 0.024162203 | 0.407984397 |
| GSM912788 | 0 | 0.013885618 | 0.00480769 | 0.094639421 | 0.120262841 | 0.02016741 | 0.015328634 | 0 | 0 | 0.1119939 | 0 | 0.28844169 | 0 | 0 | 0.000703318 | 0 | 0.00334913 | 0.017664108 | 0.308756242 |
| GSM912789 | 0.003304491 | 0.014413482 | 0.0052971 | 0.113747562 | 0.063299746 | 0.033757422 | 0.003046793 | 0 | 0 | 0.132264986 | 0 | 0.25102599 | 0.021717795 | 0 | 0 | 0 | 0.00082919 | 0.010139308 | 0.347156127 |
| GSM912790 | 0.005231132 | 0.006606741 | 0.00333173 | 0.186724065 | 0.090519733 | 0 | 0 | 0 | 0 | 0.101740387 | 0 | 0.21730824 | 0 | 0 | 0.018320516 | 0 | 0.003329203 | 0.018245273 | 0.348642985 |
| GSM912791 | 0.000315569 | 0.021374119 | 0.00604946 | 0.102994136 | 0.282849462 | 0.061291542 | 0 | 0 | 0 | 0.114181223 | 0 | 0.19854865 | 0.004274986 | 0 | 0 | 0 | 0.000749475 | 0.020198843 | 0.187172538 |
| GSM912792 | 0 | 0.009958014 | 0.00432224 | 0.12914252 | 0.028009927 | 0.035896956 | 0 | 0 | 0 | 0.162643392 | 0 | 0.21561189 | 0.001341935 | 0 | 0.021168076 | 0 | 0.000260346 | 0.011016996 | 0.38062771 |
| GSM912793 | 0 | 0.004110987 | 0.00438462 | 0.215620829 | 0.051535981 | 0 | 0.02327743 | 0 | 0 | 0.159041181 | 0 | 0.25203154 | 0 | 0 | 0.014829958 | 0 | 0.002838044 | 0.01108612 | 0.261243315 |
| GSM912794 | 0 | 0.015161632 | 0.0081536 | 0.104680708 | 0.10762976 | 0 | 0 | 0 | 0 | 0.160857417 | 0 | 0.19661241 | 0 | 0 | 0 | 0 | 0.002463623 | 0.01512079 | 0.389320061 |
| GSM912795 | 0 | 0.033356485 | 0 | 0.073300008 | 0.184843958 | 0.088954395 | 0 | 0 | 0 | 0.131660161 | 0 | 0.13455345 | 0 | 0 | 0.003089057 | 0 | 0 | 0.016591162 | 0.333651327 |
| GSM912796 | 0 | 0.011340615 | 0.00653544 | 0.056626983 | 0.073890975 | 0.018060709 | 0 | 0 | 0 | 0.097367147 | 0 | 0.34768436 | 0.013319863 | 0 | 0 | 0 | 0.000769347 | 0.0081712 | 0.366233369 |
| GSM912797 | 0 | 0.046471163 | 0.00651307 | 0.018416284 | 0.152242239 | 0 | 0 | 0 | 0 | 0.091170232 | 0 | 0.15124996 | 0.017727593 | 0.00025577 | 0 | 0 | 0.00304327 | 0.019962435 | 0.492947981 |
| GSM912798 | 0 | 0.027277442 | 0.00393416 | 0.06048688 | 0.090026172 | 0.068577594 | 0.016811097 | 0 | 0 | 0.175541372 | 0 | 0.15313503 | 0 | 0 | 0.004837963 | 0 | 0.001553894 | 0.029243344 | 0.368575052 |
| GSM912799 | 0 | 0.037034681 | 0.00712593 | 0.090548061 | 0.122246641 | 0.047964993 | 0 | 0 | 0 | 0.178711015 | 0 | 0.18963708 | 0 | 0 | 0 | 0 | 0.001916001 | 0.02217991 | 0.302635689 |
| GSM912800 | 0 | 0.013857143 | 0.00352366 | 0.059625367 | 0.129564607 | 0.05741791 | 0 | 0 | 0 | 0.089319565 | 0 | 0.18309478 | 0.00318151 | 0 | 0.002850396 | 0 | 0.001922696 | 0.011048119 | 0.444594247 |
| GSM912801 | 0 | 0.024139512 | 0.00837416 | 0.122336206 | 0.059771249 | 0 | 0.008737961 | 0 | 0 | 0.13765272 | 0 | 0.18152024 | 0 | 0 | 0.015745191 | 0 | 0.00432808 | 0.014235092 | 0.42315959 |
| GSM912802 | 0 | 0.008871723 | 0.00522519 | 0.132261915 | 0 | 0.086861401 | 0 | 0 | 0 | 0.144600971 | 0 | 0.18361617 | 0.032534936 | 0 | 0 | 0 | 3.46E-05 | 0.003652656 | 0.402340411 |
| GSM912803 | 0.001035767 | 0.005279512 | 0.00326956 | 0.125508489 | 0.076693969 | 0.025246553 | 0 | 0 | 0.016214615 | 0.111573107 | 0 | 0.16438792 | 0 | 0 | 0.019534039 | 0 | 0.001267112 | 0.015572234 | 0.434417124 |
| GSM912804 | 0 | 0.016580482 | 0.00295511 | 0.086141078 | 0.102041699 | 0.123607424 | 0 | 0 | 0 | 0.139413207 | 0.00272685 | 0.16837493 | 0 | 0 | 0 | 0 | 0.001401568 | 0.029386199 | 0.327371453 |
| GSM912805 | 0 | 0.023790413 | 0.00322151 | 0.162842746 | 0.122421338 | 0.057134513 | 0 | 0.007357388 | 0 | 0.161139097 | 0 | 0.21112808 | 0.017131726 | 0 | 0 | 0 | 0.003660596 | 0.017626192 | 0.212546401 |
| GSM912806 | 0 | 0.002353792 | 0.00380217 | 0.064810089 | 0.069892381 | 0.046047272 | 0.004143391 | 0 | 0.001358746 | 0.158603259 | 0 | 0.20942583 | 0.006054404 | 0 | 0 | 0 | 0.002734955 | 0.017690389 | 0.413083323 |
| GSM912807 | 0 | 0.025887927 | 0.00469812 | 0.116883039 | 0.078313917 | 0 | 0.002781202 | 0 | 0 | 0.082413993 | 0 | 0.25026791 | 0 | 0 | 0 | 0 | 0.004830886 | 0.001706341 | 0.432216667 |
| GSM912808 | 0 | 0.020918838 | 0.00585523 | 0.046589393 | 0.128607836 | 0.065234859 | 0 | 0 | 0 | 0.139755704 | 0 | 0.18443264 | 0.004725169 | 0 | 0 | 0 | 0 | 0.016243201 | 0.387637126 |
| GSM912809 | 0 | 0.023362874 | 0.0020218 | 0.096896071 | 0.129420676 | 0.109286118 | 0 | 0 | 0 | 0.094359605 | 0.01048801 | 0.16004289 | 0.007733005 | 0 | 0.000766481 | 0 | 0 | 0.020208446 | 0.345414017 |
| GSM912810 | 0 | 0.027294709 | 0.00894753 | 0.113960157 | 0.102957594 | 0.075814206 | 0 | 0 | 0 | 0.114245551 | 0 | 0.12947101 | 0 | 0 | 0.01563159 | 0 | 0.000893165 | 0.017146899 | 0.39363759 |
| GSM912811 | 0 | 0.014114853 | 0.00755805 | 0.103356646 | 0.099455667 | 0.047969896 | 0.003095792 | 0 | 0 | 0.129248842 | 0 | 0.19507068 | 0 | 0 | 0 | 0 | 0.002512859 | 0.014541294 | 0.383075427 |
| GSM912812 | 0 | 0.043018387 | 0 | 0.072991155 | 0.180191265 | 0.086320786 | 0.000882904 | 0 | 0 | 0.062743037 | 0 | 0.18491537 | 0 | 0 | 0.013252462 | 0 | 0.004394503 | 0.023065448 | 0.328224687 |
| GSM912813 | 0 | 0.030814617 | 0.00694946 | 0.032672945 | 0.097144674 | 0.081036804 | 0 | 0 | 0 | 0.074285408 | 0.014725098 | 0.08437949 | 0.011262013 | 0 | 0.000807174 | 0 | 0 | 0.046950508 | 0.518971804 |
| GSM912814 | 0 | 0.017661939 | 0.00158133 | 0.06716948 | 0.076754156 | 0.118442196 | 0 | 0 | 0 | 0.118606343 | 0 | 0.21562101 | 0.001123885 | 0 | 0 | 0 | 0 | 0.018772106 | 0.364267555 |
| GSM912815 | 0 | 0.025991291 | 0.00220749 | 0.178507609 | 0.149466071 | 0.04360655 | 0 | 0 | 0 | 0.180067393 | 0 | 0.17792994 | 0.015743196 | 0 | 0.00645314 | 0 | 0.00211808 | 0.015143326 | 0.202765914 |
| GSM912816 | 0.001869314 | 0.025174468 | 0 | 0.088349458 | 0.100106239 | 0.199680664 | 0 | 0 | 0 | 0.120591237 | 0 | 0.18590587 | 0.007327329 | 0 | 0.00362146 | 0 | 0.001544355 | 0.008446228 | 0.257383373 |
| GSM912817 | 0.003770348 | 0.013892609 | 0.00364078 | 0.186805099 | 0.165967837 | 0.022029009 | 0 | 0 | 0 | 0.149471375 | 0 | 0.24628437 | 0 | 0 | 0 | 0 | 0.000225038 | 0.012580741 | 0.195332803 |
| GSM912818 | 0 | 0.020556482 | 0.0018735 | 0.107146364 | 0.104756621 | 0.021586691 | 0 | 0 | 0 | 0.102160701 | 0 | 0.16252258 | 0 | 0 | 0.01374398 | 0 | 0 | 0.013430382 | 0.452222707 |
| GSM912819 | 0 | 0 | 0.0032684 | 0.25665892 | 0.080892015 | 0 | 0.009382772 | 0 | 0 | 0.218112817 | 0 | 0.2140523 | 0.007243048 | 0 | 0.006804784 | 0 | 0 | 0.015675202 | 0.187909739 |
| GSM912820 | 0 | 0.019576472 | 0.00340722 | 0.1951347 | 0.125766504 | 0.019622315 | 0 | 0 | 0 | 0.086900456 | 0 | 0.20384235 | 0.013591741 | 0 | 0 | 0 | 0.001301078 | 0.006476209 | 0.324380949 |
| GSM912821 | 0 | 0.012666368 | 0.00499942 | 0.173586598 | 0.067619722 | 0 | 0 | 0 | 0 | 0.164368563 | 0 | 0.21187362 | 0.024704754 | 0 | 0 | 0 | 0.002543055 | 0 | 0.3376379 |
| GSM912822 | 0.007539019 | 0.011326462 | 0 | 0.161857606 | 0.135670829 | 0.034782372 | 0 | 0 | 0 | 0.094885008 | 0 | 0.17098701 | 0 | 0 | 0.01456134 | 0 | 0.004596771 | 0.017017188 | 0.346776399 |
| GSM912823 | 0.005537126 | 0.008699111 | 0.00250389 | 0.139714548 | 0.087617295 | 0 | 0.002511302 | 0 | 0 | 0.119789822 | 0 | 0.31288345 | 0.008939685 | 0 | 0 | 0 | 0.002737968 | 0.005030335 | 0.304035469 |
| GSM912824 | 0 | 0.03064477 | 0.00451701 | 0.161984341 | 0.184471718 | 0 | 0 | 0 | 0 | 0.128921656 | 0 | 0.22801868 | 0.002752191 | 0 | 0.000953445 | 0 | 0 | 0.008406964 | 0.249329226 |
| GSM912825 | 0 | 0.017670575 | 0.00515356 | 0.145295891 | 0.106840612 | 0.036310629 | 0 | 0 | 0 | 0.148269574 | 0 | 0.17013751 | 0 | 0 | 0.003924713 | 0 | 0.005901011 | 0.013958549 | 0.346537374 |
| GSM912826 | 0 | 0.010655229 | 0.00294886 | 0.092159309 | 0.048863231 | 0.064738141 | 0 | 0 | 0 | 0.11957186 | 0 | 0.36955223 | 0 | 0 | 0 | 0 | 0.002124118 | 0.019686142 | 0.26970088 |
| GSM912827 | 0 | 0.022563865 | 0.00170391 | 0.235592572 | 0.135083617 | 0 | 0.00359057 | 0 | 0 | 0.14873342 | 0 | 0.25405341 | 0.014641493 | 0 | 0 | 0 | 0.002789458 | 0.003997709 | 0.177249972 |
| GSM912828 | 0 | 0.005788943 | 0.00413864 | 0.22452463 | 0.073625669 | 0 | 0 | 0 | 0 | 0.17410552 | 0 | 0.23890062 | 0.017659915 | 0 | 0 | 0 | 0.003504289 | 0.012322952 | 0.245428821 |
| GSM912829 | 0 | 0.015400964 | 0.00491506 | 0.071560805 | 0.079090404 | 0 | 0 | 0 | 0 | 0.103890222 | 0 | 0.15732671 | 0 | 0 | 0.007342136 | 0 | 0.003221605 | 0.015585621 | 0.541666468 |
| GSM912830 | 0 | 0.012500412 | 0.00475292 | 0.102325165 | 0.082287384 | 0.021757767 | 0 | 0 | 0 | 0.14537951 | 0 | 0.11368644 | 0.006310549 | 0 | 0 | 0 | 3.88E-05 | 0.003831108 | 0.507130001 |
| GSM912831 | 0 | 0.013526884 | 0.00272047 | 0.085837111 | 0.123251472 | 0.0334489 | 0.002112249 | 0 | 0 | 0.134751516 | 0 | 0.20583832 | 0 | 0 | 0.0006561 | 0 | 0.00197448 | 0.010551071 | 0.385331428 |
| GSM912832 | 0 | 0.002586265 | 0.00598224 | 0.092007048 | 0.149605912 | 0.060541577 | 0 | 0 | 0 | 0.064316713 | 0.001176751 | 0.21018761 | 0 | 0 | 0.007946188 | 0 | 0.000796807 | 0.035740599 | 0.369112291 |
| GSM912833 | 0 | 0.016524127 | 0.0093887 | 0.172704872 | 0.143120247 | 0.036449091 | 0 | 0 | 0 | 0.092289599 | 0 | 0.21171718 | 0 | 0 | 0.026175358 | 0 | 0.002731536 | 0.009112257 | 0.279787031 |
| GSM912834 | 0 | 0.028397213 | 0 | 0.1189687 | 0.190136804 | 0.092746846 | 0 | 0 | 0 | 0.070515589 | 0.002057502 | 0.14681347 | 0 | 0 | 0.017859465 | 0 | 0.001687771 | 0.010092376 | 0.320724265 |
| GSM912835 | 0.003229609 | 0.049461805 | 0 | 0.0830811 | 0.145194896 | 0.125940063 | 0 | 0 | 0.017987472 | 0.079204754 | 0 | 0.15819182 | 0 | 0 | 0.027537702 | 0 | 0.003349655 | 0.014935654 | 0.291885466 |
| GSM912836 | 0 | 0.0247819 | 0 | 0.039907781 | 0.180668495 | 0.139506589 | 0 | 0 | 0 | 0.113726323 | 0 | 0.16432924 | 0 | 0 | 0 | 0 | 0.00203523 | 0.025196406 | 0.309848035 |
| GSM912837 | 0.007099996 | 0.016075636 | 0 | 0.087650833 | 0.153763104 | 0.111695844 | 0 | 0 | 0 | 0.079920147 | 0.002331574 | 0.18490728 | 0 | 0 | 0 | 0 | 0.002431764 | 0.030214239 | 0.32390958 |
| GSM912838 | 0.010037584 | 0 | 0.00018084 | 0.299659906 | 0.101614955 | 0 | 0.00384287 | 0 | 0 | 0.225463626 | 0 | 0.16721659 | 0 | 0 | 0 | 0 | 0 | 0.015108931 | 0.176874695 |
| GSM912839 | 0.009349114 | 0.018345828 | 0 | 0.206835942 | 0.024791461 | 0.074165398 | 0 | 0 | 0 | 0.203912854 | 0 | 0.1962683 | 0 | 0 | 0 | 0 | 0.002160676 | 0.003466209 | 0.260704219 |
| GSM912840 | 0 | 0.035709368 | 0.00561464 | 0.081865814 | 0.175602521 | 0.023452785 | 0 | 0.011259861 | 0 | 0.135687685 | 0 | 0.14586949 | 0.001635923 | 0.001587728 | 0 | 0 | 0.000698686 | 0.017774454 | 0.363241044 |
| GSM912841 | 0.00900341 | 0.005001042 | 0.00329313 | 0.154130225 | 0.106435888 | 0.070785161 | 0 | 0 | 0 | 0.161941351 | 0.004456323 | 0.18098268 | 0 | 0 | 0 | 0 | 0.001398721 | 0.028912666 | 0.273659402 |
| GSM912842 | 0.007815779 | 0.051706208 | 0 | 0.078470272 | 0.102676172 | 0.013616427 | 0 | 0 | 0 | 0.10754744 | 0 | 0.08867547 | 0 | 0.000100974 | 0 | 0 | 0.002446701 | 0.024418721 | 0.522525837 |
| GSM912843 | 0.01805959 | 0.008991 | 0.00153367 | 0.134833486 | 0.13810776 | 0.066851269 | 0 | 0 | 0 | 0.178874266 | 0.001586872 | 0.1642187 | 0 | 0 | 0 | 0 | 0 | 0.023004039 | 0.263939345 |
| GSM912844 | 0 | 0.012247485 | 0.00274124 | 0.071474514 | 0.07906909 | 0.084039283 | 0.002379414 | 0 | 0 | 0.099678654 | 0 | 0.21107809 | 0.011932729 | 0 | 0 | 0 | 0 | 0.014528438 | 0.410831068 |
| GSM912845 | 0 | 0.02560659 | 0.00963266 | 0.053015757 | 0.167287486 | 0.053227828 | 0 | 0 | 0 | 0.11167983 | 0.016551286 | 0.18164032 | 0 | 0 | 0 | 0 | 0.008850856 | 0.016080405 | 0.356426974 |
| GSM912846 | 0.004974228 | 0.012289012 | 0.0008547 | 0.04025312 | 0.159162568 | 0.088940771 | 0.005005398 | 0 | 0 | 0.100083581 | 0 | 0.17532001 | 0 | 0 | 0 | 0 | 0.00202609 | 0.014197974 | 0.396892548 |
| GSM912847 | 0.003111836 | 0.012021487 | 0.00043915 | 0.052991444 | 0.088721549 | 0.051303954 | 0 | 0 | 0 | 0.142593203 | 0 | 0.18604481 | 0.008359709 | 0 | 0 | 0 | 0.001423677 | 0.005552847 | 0.447436334 |
| GSM912848 | 0.004739088 | 0.017071683 | 0 | 0.069755539 | 0.057599858 | 0.155890555 | 0 | 0 | 0 | 0.149383856 | 0 | 0.22479588 | 0 | 0 | 0 | 0 | 0.002365749 | 0.025254094 | 0.293143692 |
| GSM912849 | 0.007549399 | 0.010187566 | 0.00256339 | 0.087623747 | 0.062980523 | 0.130310981 | 0 | 0.002722708 | 0 | 0.176434421 | 0 | 0.18066804 | 0 | 0 | 0 | 0 | 0 | 0.019976569 | 0.31898265 |
| GSM912850 | 0 | 0.010615823 | 0.00993496 | 0.059176808 | 0.113278281 | 0.042775195 | 0 | 0 | 0 | 0.126084213 | 0 | 0.12998305 | 0 | 0.000581286 | 0 | 0 | 0 | 0.039963834 | 0.467606547 |
| GSM912851 | 0.00331209 | 0.011956956 | 0.00231564 | 0.120184796 | 0.077074686 | 0.084765891 | 0.012172996 | 0 | 0 | 0.151590315 | 0 | 0.22385652 | 0 | 0 | 0 | 0 | 0.00175161 | 0.015279261 | 0.295739245 |
| GSM912852 | 0 | 0.013431925 | 0.00198425 | 0.151498345 | 0.066743892 | 0.05230237 | 0 | 0 | 0 | 0.192404484 | 0 | 0.1931568 | 0.002480598 | 0 | 0 | 0 | 0.001915617 | 0.001862947 | 0.322218773 |
| GSM912853 | 0 | 0.012390233 | 0.00286586 | 0.121005985 | 0.130411701 | 0.065700194 | 0 | 0 | 0 | 0.157989718 | 0 | 0.18215756 | 0 | 0 | 0 | 0 | 0 | 0.010433621 | 0.317045133 |
| GSM912854 | 0.007274766 | 0.011598823 | 0.00107623 | 0.126899705 | 0.100883493 | 0.002375314 | 0.008242688 | 0 | 0 | 0.129947657 | 0 | 0.16055714 | 0 | 0.002420352 | 0 | 0 | 0 | 0.023834668 | 0.424889161 |
| GSM912855 | 0 | 0.016501121 | 0.0006443 | 0.201769757 | 0.002441824 | 0.129226451 | 0.006900739 | 0 | 0 | 0.204038134 | 0 | 0.17054851 | 0.000983615 | 0 | 0 | 0 | 0.000922239 | 0.003143501 | 0.262879813 |
| GSM912856 | 0.005411357 | 0.017287554 | 0 | 0.038336159 | 0.159637773 | 0.148355806 | 0 | 0 | 0 | 0.128594821 | 0 | 0.17622779 | 0 | 0 | 0 | 0 | 0.003183432 | 0.027280601 | 0.295684705 |
| GSM912857 | 0.008098502 | 0.014044494 | 0 | 0.120970376 | 0.105297155 | 0.131093444 | 0 | 0 | 0 | 0.089194865 | 0.001344465 | 0.17235989 | 0 | 0 | 0 | 0 | 0.003980364 | 0.032611657 | 0.321004789 |
| GSM912858 | 0.002205954 | 0 | 0.00309672 | 0.304964362 | 0.121743849 | 0.00249024 | 0 | 0 | 0 | 0.231926668 | 0 | 0.15964308 | 0.000711282 | 0 | 0 | 0 | 0 | 0 | 0.173217847 |
| GSM912859 | 0.003791549 | 0.013977886 | 8.60E-05 | 0.222045662 | 0.033448678 | 0.060480136 | 0 | 0 | 0 | 0.204711285 | 0 | 0.19663944 | 0 | 0 | 0 | 0 | 0.003131935 | 0.003076827 | 0.258610572 |
| GSM912860 | 0 | 0.029556391 | 0.00427191 | 0.099084464 | 0.161980371 | 0.021507261 | 0 | 0.009834161 | 0 | 0.123790985 | 0 | 0.14672233 | 0.00474729 | 0.001262138 | 0 | 0 | 0.001868248 | 0.030425736 | 0.364948723 |
| GSM912861 | 0.00594866 | 0.005157615 | 0.00313343 | 0.153263848 | 0.127520055 | 0.059744222 | 0 | 0 | 0 | 0.177470901 | 0 | 0.17752993 | 0 | 0 | 0 | 0 | 0 | 0.016955883 | 0.273275451 |
| GSM912862 | 0 | 0.041430741 | 0 | 0.112679646 | 0.088620697 | 0.018866863 | 0 | 0 | 0 | 0.114333374 | 0 | 0.08897613 | 0 | 0.000449555 | 0 | 0 | 0 | 0.017787649 | 0.516855344 |
| GSM912863 | 0.003591604 | 0.014833884 | 0 | 0.161548568 | 0.054783619 | 0.10358025 | 0 | 0 | 0 | 0.178295382 | 0.004808918 | 0.17797857 | 0 | 0 | 0 | 0 | 0.001004752 | 0.025717384 | 0.27385707 |
| GSM912864 | 0 | 0.011424602 | 0.00187989 | 0.071448611 | 0.076934996 | 0.085886256 | 0.002839492 | 0 | 0 | 0.092095873 | 0 | 0.20296173 | 0.001809496 | 0 | 0 | 0 | 0 | 0.01564021 | 0.437078839 |
| GSM912865 | 0 | 0.034532898 | 0.00536783 | 0.041772619 | 0.154302076 | 0.058531623 | 0 | 0 | 0 | 0.135055846 | 0 | 0.20423708 | 0 | 0 | 0 | 0 | 0.006578031 | 0.021880498 | 0.337741498 |
| GSM912866 | 0.007044421 | 0.013683957 | 0 | 0.040218813 | 0.127017992 | 0.121390973 | 0.003148348 | 0 | 0 | 0.112049465 | 0 | 0.17259083 | 0 | 0 | 0 | 0 | 0.000521326 | 0.013526938 | 0.388806932 |
| GSM912867 | 0.000712805 | 0.011211568 | 0.00013668 | 0.057041367 | 0.083825334 | 0.077114911 | 0 | 0 | 0 | 0.139693246 | 0 | 0.18514025 | 0.006695955 | 0 | 0 | 0 | 0.000546295 | 0.000298774 | 0.43758281 |
| GSM912868 | 0.001242733 | 0.009395642 | 0.00099936 | 0.065991719 | 0.053593372 | 0.173208727 | 0 | 0 | 0 | 0.146309888 | 0 | 0.2319815 | 0 | 0 | 0 | 0 | 0.002295226 | 0.021079102 | 0.293902728 |
| GSM912869 | 0.004440541 | 0.011873019 | 0.00249123 | 0.083889449 | 0.070195704 | 0.142699725 | 0 | 0 | 0 | 0.16391174 | 0 | 0.16935573 | 0 | 0 | 0 | 0 | 0.000497727 | 0.01761609 | 0.333029048 |
| GSM912870 | 0 | 0.017943276 | 0.00617294 | 0.040357651 | 0.131341502 | 0.053651487 | 0 | 0 | 0 | 0.127365809 | 0 | 0.15147079 | 0 | 0 | 0 | 0 | 0 | 0.012801478 | 0.45889506 |
| GSM912871 | 0.003136292 | 0.01122608 | 0.00055922 | 0.089133167 | 0.059211082 | 0.124526918 | 0.014052024 | 0 | 0 | 0.154453583 | 0 | 0.22552467 | 0 | 0 | 0 | 0 | 0.003463585 | 0.011286232 | 0.303427146 |
| GSM912872 | 0 | 0.012849788 | 0.00195469 | 0.155033245 | 0.074697121 | 0.034094308 | 0.009322319 | 0 | 0 | 0.18110639 | 0 | 0.17914076 | 0 | 0 | 4.96E-05 | 0 | 0.001399331 | 0.000236404 | 0.350116053 |
| GSM912873 | 0 | 0.015334062 | 1.72E-05 | 0.127591649 | 0.108874263 | 0.09390195 | 0 | 0 | 0 | 0.160976463 | 0 | 0.18077034 | 0 | 0 | 0.000134256 | 0 | 0.00180683 | 0.005301815 | 0.305291166 |
| GSM912874 | 0 | 0.011594412 | 0.00357044 | 0.141486556 | 0.091500578 | 0.014685002 | 0 | 0 | 0 | 0.123658476 | 0 | 0.15463947 | 0 | 0 | 0.003444417 | 0 | 0 | 0.016502462 | 0.438918194 |
| GSM912875 | 0 | 0.002801385 | 0.0064906 | 0.209741721 | 0.031938995 | 0.084949784 | 0 | 0 | 0 | 0.189015265 | 0 | 0.22538382 | 0.010771546 | 0 | 0 | 0 | 0 | 0 | 0.238906888 |
|  |  |  |  |  |  |  |  |  |  |  |  |  |  |  |  |  |  |  |  |
