# Supplementary material for: APC and ZBTB2 May Mediate M2 Macrophage Infiltration to Promote the Development of Renal Fibrosis: A Bioinformatics Analysis
Source: Biomed Res Int. 2024 Sep 18;2024:5674711. doi: 10.1155/2024/5674711 (PMC11424844; doi:10.1155/2024/5674711)
Supplement: Supporting Information 2 — Figure S1: PCA and standardized analysis. (A) shows the PCA results of the two groups of samples, and (B) shows the histogram after sample normalization. Figure S2: sample clustering diagram. Figure S3: sample dendrogram and feature heat map demonstrating the correlation between samples and immune infiltrating cells. Figure S4: scale-free network test and choice of soft threshold. (A) is a scale-free network test, and (B) shows the choice of soft threshold. [file 5674711.f2.docx]

**Supplementary Figure 1** PCA and standardized analysis


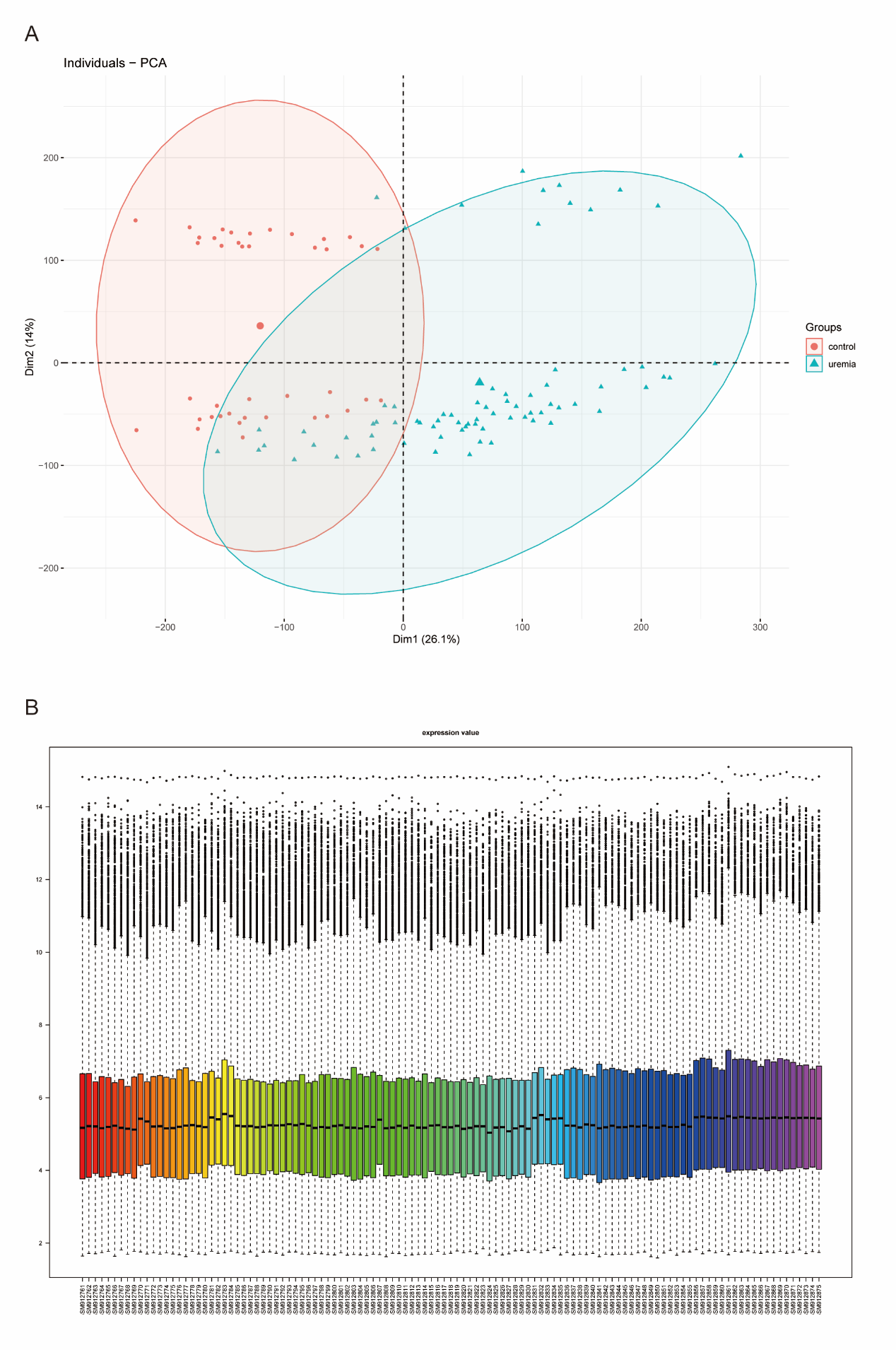


**(**A) shows the PCA results of the two groups of samples, and (B) shows the histogram after sample normalization.

**Supplementary Figure 2** sample clustering diagram.


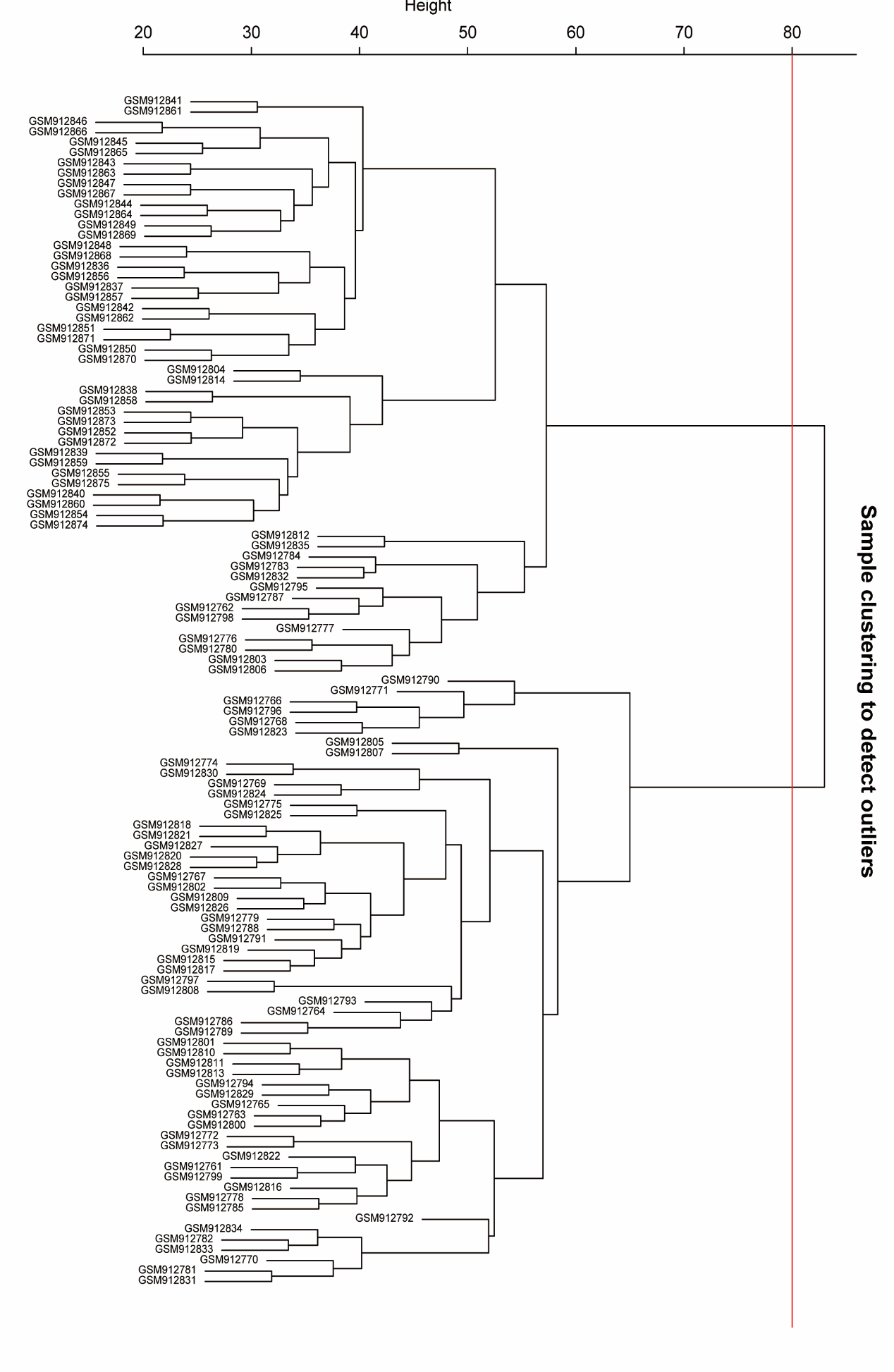


**Supplementary Figure 3 s**ample dendrogram and feature heatmap demonstrating the correlation between samples and immune infiltrating cells.
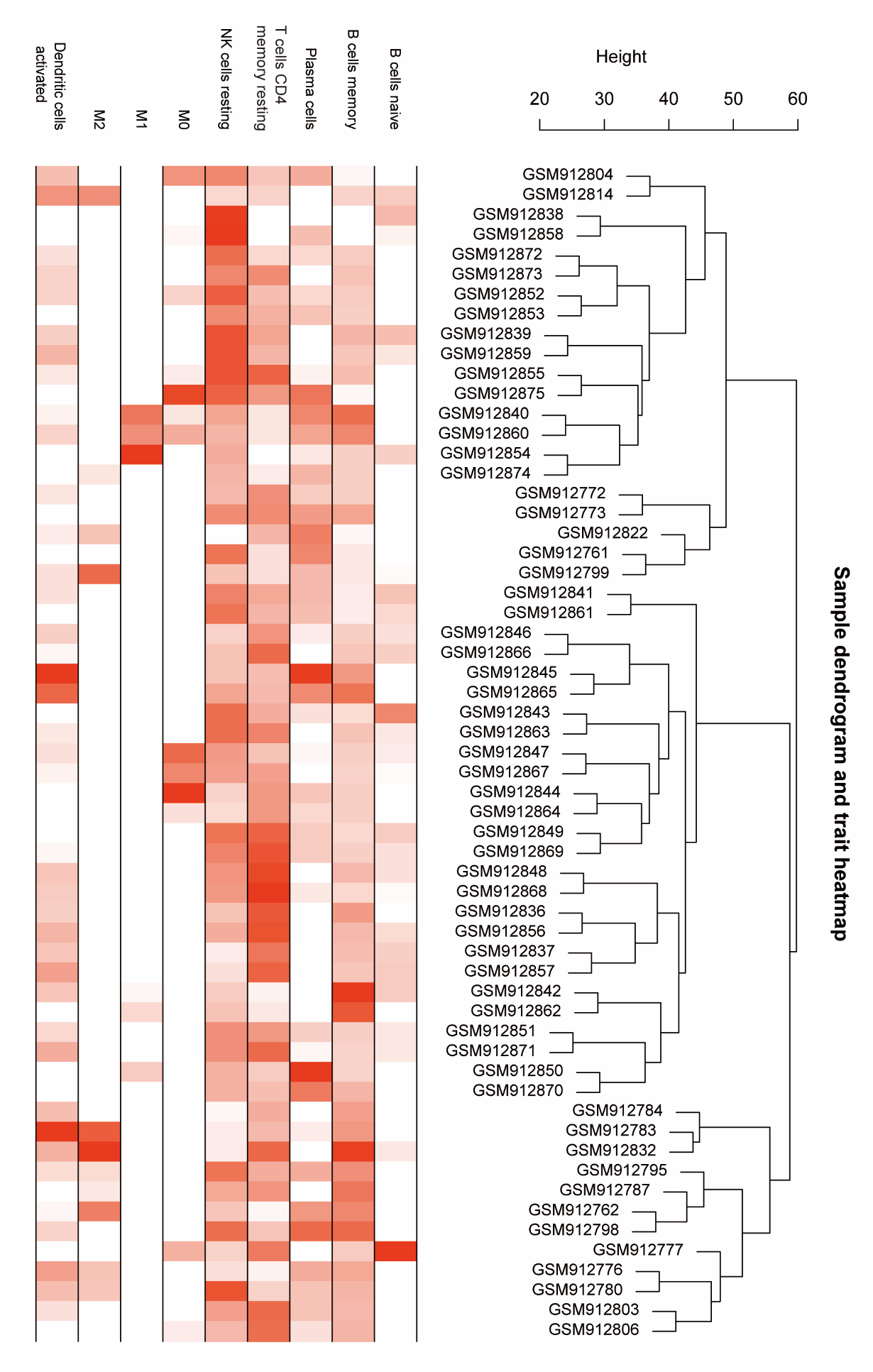


**Supplementary Figure 4** scale-free network test and choice of soft threshold.
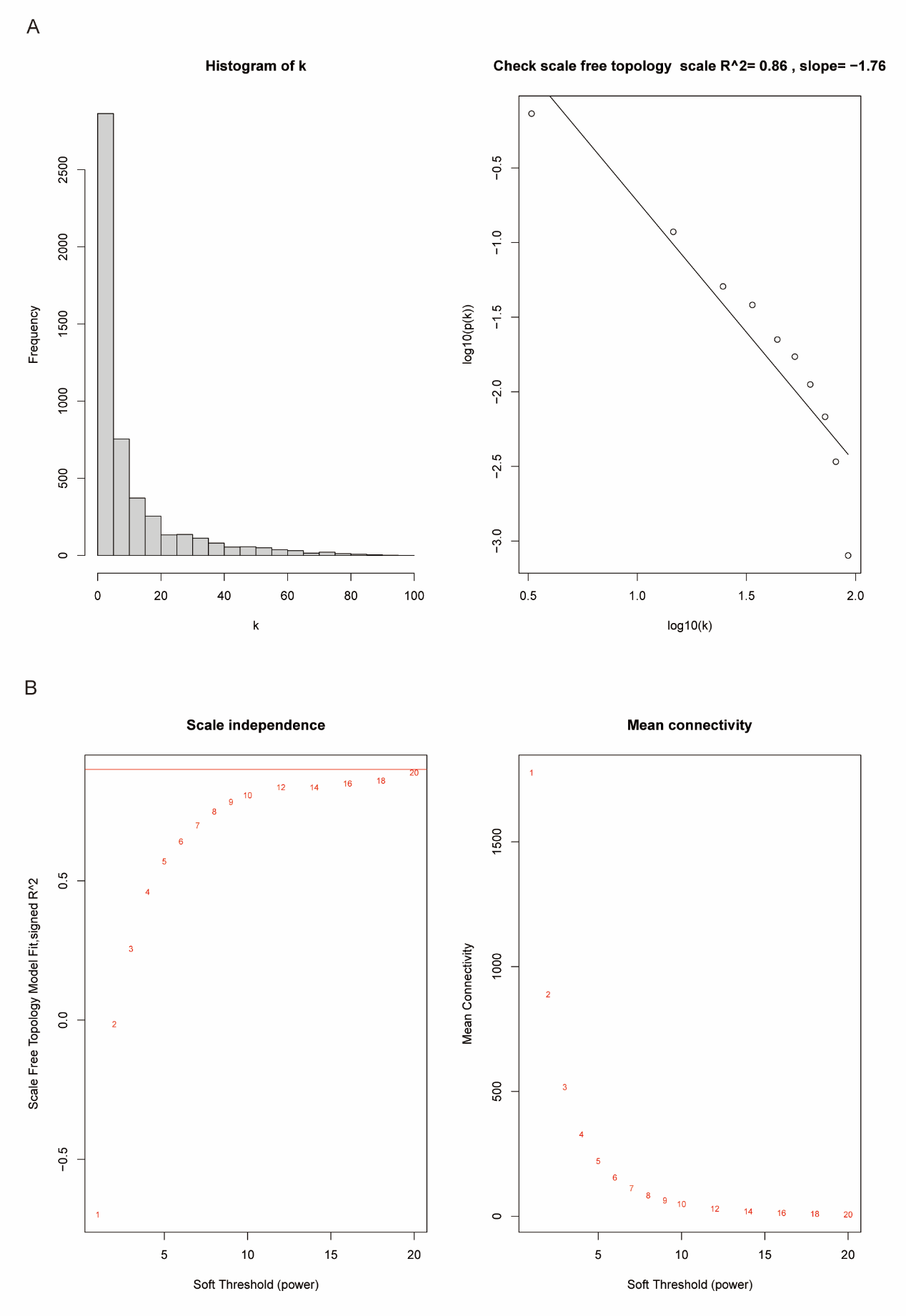


(A) is a scale-free network test, (B) shows the choice of soft threshold.
